# The effect of extracellular matrix on the precision medicine utility of pancreatic cancer patient–derived organoids

**DOI:** 10.1172/jci.insight.172419

**Published:** 2024-01-09

**Authors:** Jan C. Lumibao, Shira R. Okhovat, Kristina L. Peck, Xiaoxue Lin, Kathryn Lande, Shira Yomtoubian, Isabella Ng, Hervé Tiriac, Andrew M. Lowy, Jingjing Zou, Dannielle D. Engle

**Affiliations:** 1 Salk Institute for Biological Studies, La Jolla, California, USA.; 2 Department of Surgery, Division of Surgical Oncology, Moores Cancer Center, and; 3 Division of Biostatistics and Bioinformatics, Herbert Wertheim School of Public Health and Human Longevity Science, UCSD, San Diego, California, USA.

**Keywords:** Cell Biology, Oncology, Cancer, Drug therapy, Extracellular matrix

## Abstract

The use of patient-derived organoids (PDOs) to characterize therapeutic sensitivity and resistance is a promising precision medicine approach, and its potential to inform clinical decisions is now being tested in several large multiinstitutional clinical trials. PDOs are cultivated in the extracellular matrix from basement membrane extracts (BMEs) that are most commonly acquired commercially. Each clinical site utilizes distinct BME lots and may be restricted due to the availability of commercial BME sources. However, the effect of different sources of BMEs on organoid drug response is unknown. Here, we tested the effect of BME source on proliferation, drug response, and gene expression in mouse and human pancreatic ductal adenocarcinoma (PDA) organoids. Both human and mouse organoids displayed increased proliferation in Matrigel compared with Cultrex and UltiMatrix. However, we observed no substantial effect on drug response when organoids were cultured in Matrigel, Cultrex, or UltiMatrix. We also did not observe major shifts in gene expression across the different BME sources, and PDOs maintained their classical or basal-like designation. Overall, we found that the BME source (Matrigel, Cultrex, UltiMatrix) does not shift PDO dose-response curves or drug testing results, indicating that PDO pharmacotyping is a robust approach for precision medicine.

## Abstract 2

<p>Different commercial basement membrane extracts affect organoid growth but do not markedly influence organoid drug response.</p>

## Introduction

The 5-year relative survival rate for patients with pancreatic ductal adenocarcinoma (PDA) is 12% (1). As the incidence of PDA continues to rise (2), strategies to increase survival are imperative. Surgical resection remains the only curative option. However, most patients are ineligible due to the presence of metastasis at the time of diagnosis (3). Standard-of-care chemotherapeutic regimens include gemcitabine/nab-paclitaxel and FOLFIRINOX, which demonstrate median survival times of 8.5 and 11.1 months, respectively (4, 5). Furthermore, since heterogeneity in tumor biology and treatment response continues to be revealed, personalized medicine approaches are becoming increasingly necessary.

The use of patient-derived organoids (PDOs) has the potential to revolutionize care for patients with PDA (6). PDOs are 3D cultures in defined conditions that support propagation of normal, premalignant, and neoplastic cells from primary tissue (7–11). Organoid technology has emerged as a promising avenue for precision medicine (12). The ability to derive PDA PDOs from surgical resections, rapid autopsies (RAP), and endoscopic ultrasound–guided fine-needle biopsies allows for a broad sampling of patients with PDA to encompass inter- and intrapatient tumor heterogeneity (8). Importantly, PDA PDOs mirror patient tumor genetics, gene expression, and treatment response, situating them as promising tools for more precision medicine efforts to identify alternative treatment strategies (8, 13–15).

Since the use of organoids has expanded and become more accessible, variations in culture conditions have been introduced to optimize PDA PDO generation and growth. A number of studies have delineated the effect of the liquid media composition on organoid phenotype, transcriptome, and drug response (16, 17). Commercial products to support organoid studies have become more widespread and include a wide variety of basement membrane extracts (BME), which serve as 3D scaffolds. Recent studies have described the use of fully synthetic and defined hydrogels to support organoids of colon, bile duct, and mammary gland origin (18–21). However, BMEs derived from Engelbreth-Holm-Swarm (EHS) murine sarcomas, most notably Matrigel, but also products such as Cultrex and UltiMatrix, remain the most widely used (19). EHS tumor–derived BMEs contain heterogenous mixtures of extracellular matrix proteins, primarily laminins, collagen IV, entactin, and perlecan as well as tumor-derived proteins and growth factors, which contribute to batch-to-batch variability (18, 19). Regardless, these BMEs have historically served as the scaffold for organoid culture. However, the effect of different EHS tumor–derived BMEs on organoid growth, chemosensitivity, and global gene expression remains unclear. Importantly, previous reports have demonstrated the clinical utility and relevance of PDA PDO pharmacotyping using Matrigel (8, 13, 15). These studies and others have prompted the design of clinical trials based on the use of organoid-guided chemotherapy. However, due to the COVID-19 pandemic, severe supply chain issues have limited BME availability. These issues range from complete inability to secure the same commercial source of BME or dramatic reduction of available BME within the same lot. The effect of the commercial source and lot of BME on drug response and prognostic gene expression programs has not been explored yet is critical for the deployment of PDO clinical trials.

Here, we report the effect of BME on PDA organoid growth, response to standard-of-care chemotherapy as well as targeted therapy, and gene expression patterns. While we find that BME source has a significant and substantial effect on organoid growth, drug response and gene expression remain consistent across multiple lots and commercial sources. Results from this study provide insight into selecting BME for organoid culture and for characterizing PDO sensitivity and resistance to both chemotherapies and targeted therapies, which may assist in guiding clinical decisions.

## Results

### BME effects mouse and human organoid growth.

**Figure 1:**
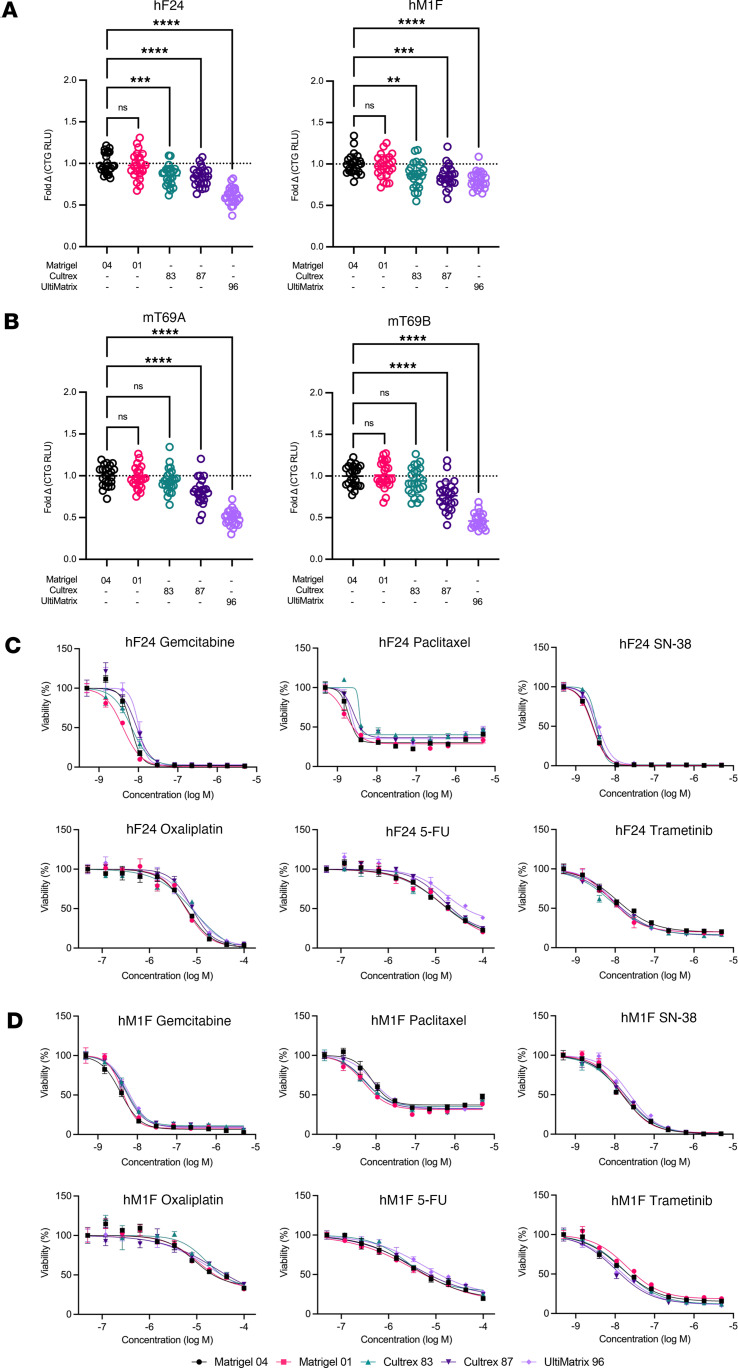
Basement membrane extract affects growth of mouse and human organoids but does not significantly shift dose-response curves. (**A**) Growth of patient-derived organoids hF24 and hM1F cultured in Matrigel 04, Matrigel 01, Cultrex 83, Cultrex 87, or UltiMatrix 96 for 5 days as determined by levels of intracellular ATP (Cell Titer Glo [CTG]). Data represent mean ± SD. Statistical significance was determined by 1-way ANOVA. **P ≤ 0.01, ***P ≤ 0.001, ****P ≤ 0.0001. (**B**) Growth of mouse organoids mT69A and mT69B cultured in Matrigel 04, Matrigel 01, Cultrex 83, Cultrex 87, or UltiMatrix 96 for 3 days as determined by levels of intracellular ATP (CTG). Data represent mean ± SD. Statistical significance was determined by 1-way ANOVA. ****P ≤ 0.0001. (**C**) Dose-response curves for hF24 treated with gemcitabine, paclitaxel, SN-38, oxaliplatin, 5-FU, or trametinib during culture in Matrigel 04, Matrigel 01, Cultrex 83, Cultrex 87, or UltiMatrix 96. Data represent mean ± SD of triplicate values fitted with a 4-parameter log-logistic function. (**D**) Dose-response curves for hM1F treated with gemcitabine, paclitaxel, SN-38, oxaliplatin, 5-FU, and trametinib during culture in Matrigel 04, Matrigel 01, Cultrex 83, Cultrex 87, or UltiMatrix 96. Data represent mean ± SD of triplicate values fitted with a 4-parameter log-logistic function.

To investigate the influence of BME on organoid growth, human and mouse PDA organoids (Supplemental Tables 1 and 2; supplemental material available online with this article; https://doi.org/10.1172/jci.insight.172419DS1) were cultivated in Matrigel 04 and then plated in either Matrigel 04, Matrigel 01, Cultrex 83, Cultrex 87, or UltiMatrix 96. Total protein concentration of each BME ranged from 7.9 to 11.6 mg/mL, with Matrigel 04 exhibiting the lowest protein concentration and UltiMatrix 96 containing the highest (Supplemental Figure 1A). Intracellular ATP levels were measured after 4 (mouse) or 6 days of culture (human) (Supplemental Figure 1B). Human PDOs and mouse organoids exhibited reduced growth in Cultrex and UltiMatrix compared with Matrigel (Figure 1, A and B, and Supplemental Figure 2). Mouse organoid proliferation did not recover even after multiple passages in Cultrex, suggesting that this growth deficit is not due to a lack of adaptation period (data not shown). In most experimental replicates across mouse and human organoids, there was no difference in proliferation between Matrigel lots. In 1 of 3 experiments, there was a small but significant decrease (<10%) in growth of hM1F and hF24 in Matrigel 01 compared with Matrigel 04 (Supplemental Figure 2, A and B). Similarly, 1 of 2 experiments demonstrated a significant decrease (24%) in hT1 growth in Matrigel 01 (Supplemental Figure 2C). Mouse organoid mT69B consistently exhibited no significant differences in intracellular ATP levels between Matrigel lots (*n* = 4 of 4), while mT69A exhibited 17-23% changes between Matrigel lots (*n* = 2 of 4) (Supplemental Figure 2, D and E). In nearly all hPDO experiments, there was a significant, and sometimes substantial (e.g., 27% in Cultrex 83 relative to Matrigel 04), reduction in growth when cultured in either lot of Cultrex or UltiMatrix compared with Matrigel 04 (Figure 1A and Supplemental Figure 2, A and B). Mouse organoids exhibited more mixed results across experimental repeats but consistently (*n* = 3 of 4 mT69A, *n* = 4 of 4 mT69B) exhibited significant, and sometimes substantial (e.g., 55% decrease in UltiMatrix 96 relative to Matrigel 04), decreased growth in UltiMatrix 96 (Figure 1B and Supplemental Figure 2, D and E). In the setting of Cultrex, mouse organoid proliferation trended slower relative to Matrigel. mT69A and mT69B grew significantly slower in Cultrex 83 (A: *n* = 2 of 4, B: *n* = 2 of 4) and 87 (A: *n* = 3 of 4, B: *n* = 2 of 4), while exhibiting no significant difference relative to Matrigel 04 in 25%–50% of the experiments. These data collectively indicate that mouse and human organoids grow significantly more in Matrigel compared with Cultrex or UltiMatrix. The effect of BME source on PDO expansion may be problematic for completion of drug testing for patients within a clinically relevant timeline.

In addition to expansion of established organoids, we sought to assess the effect of BME on initial organoid generation efficiency. We first addressed this in the mouse setting using pancreata from *LSL-Kras^G12D/+^; LSL-Trp53^R172H/+^;Pdx-1-Cre* (KPC) mice. Tissue specimens were dissociated and evenly distributed among Matrigel 04, Matrigel 01, Cultrex 83, Cultrex 87, and UltiMatrix 96. There were no differences noted in absolute take rate among the different BMEs in 2 pancreata that harbored preinvasive lesions (A5462 and A5504) and 1 pancreatic tumor with mixed adenocarcinoma and sarcomatoid histopathology (A5505) (Supplemental Figure 3A). However, A5462 produced larger organoids with greater cross-sectional area by day 3 in Matrigel compared with Cultrex and UltiMatrix (Supplemental Figure 3B). After the first passage, organoids expanded with greater efficiency in Matrigel compared with Cultrex and UltiMatrix BMEs, necessitating passaging cultures in Matrigel 3 days in advance of those in Cultrex and UltiMatrix. A5504 and A5505 both yielded organoids in all 5 BMEs by day 4, and organoids were expanded efficiently in all conditions (Supplemental Figure 3, C and D). No differences in culture schedule were noted, since cultures were passaged in order to maintain BME integrity rather than due to confluence.

In the human setting, received clinical specimens were often characterized by a paucity of cellular material and a scarcity of bulk material, which undermined organoid generation efficiency across 5 conditions. Alternatively, tissue from a patient wedge biopsy of a PDA liver metastatic lesion was plated only in Matrigel 01, yielding visible organoids by day 4 (Supplemental Figure 3E). After 3 passages, these confirmed human organoids were distributed across the 5 BME conditions, exhibiting sufficient viability and quantity in all lots to support expansion of these early-passage PDOs, with slightly more organoids observed in Matrigel (Supplemental Figure 3E). Overall, these results indicate that organoid generation from both mouse and human tissue specimens is feasible in each BME tested, with early-phase expansion often occurring most quickly in Matrigel.

### BME has minimal effects on drug response.

To investigate the influence of BME on drug response, we measured the dose response of both mouse and human organoids to standard-of-care chemotherapies: gemcitabine, paclitaxel, SN-38 (active metabolite of irinotecan), oxaliplatin, and 5-fluorouracil (5-FU) as well as the targeted small molecule trametinib (third-generation MEK inhibitor) (Supplemental Figure 1B). Organoids were allowed to reform and then subjected to pharmacotyping. We found that the dose-response curves for gemcitabine, SN-38, and trametinib across all BMEs were largely overlapping in both hF24 and hM1F (*n* = 3 of 3) (Figure 1, C and D, and Supplemental Figure 4, A and B). The dose-response curves in hT1 were also consistent across BMEs for SN-38 (*n* = 2 of 2) but exhibited some variability for gemcitabine (*n* = 1 of 2) and trametinib (*n* = 1 of 2) between Matrigel, Cultrex, and UltiMatrix (Supplemental Figure 4C). Paclitaxel dose-response curves across all BMEs were also largely overlapping in hM1F (*n* = 3 of 3) (Figure 1D), hT1 (*n* = 2 of 2) (Supplemental Figure 4C), and hF24 (*n* = 2 of 3) (Figure 1C and Supplemental Figure 4A). Oxaliplatin also displayed consistent dose-response curves, regardless of BME in hF24 (*n* = 3 of 3), hM1F (*n* = 2 of 3), and hT1 (*n* = 2 of 2) (Figure 1, C and D, and Supplemental Figure 4, A–C). Additionally, 5-FU exhibited overlapping dose-response curves in hF24 (*n* = 3 of 3) (Figure 1C and Supplemental Figure 4A) and hM1F (*n* = 2 of 3) (Figure 1D and Supplemental Figure 4B). However, we observed more variability in dose-response curves for 5-FU in hT1 as a function of BME in both experimental repeats conducted (Supplemental Figure 4C).

Mouse organoids were less sensitive to all agents tested relative to hPDOs, with oxaliplatin and 5-FU dose-response curves failing to reach 50% cytotoxicity (Supplemental Figure 4, D and E). Gemcitabine and SN-38 mouse organoid dose-response curves exhibited no consistent or substantial shifts when cultured in different BMEs (Supplemental Figure 4, D and E). Dose-response curves for trametinib were also largely overlapping, regardless of BME for both mT69A (*n* = 3 of 4) and mT69B (*n* = 3 of 4), although both organoid lines were more resistant in 1 of 4 experimental replicates when cultured in UltiMatrix 96 (Supplemental Figure 4, D and E). We observed a similar dose-response shift for paclitaxel when mouse organoids were cultured in UltiMatrix (*n* = 1 of 4) (Supplemental Figure 4, D and E). Overall, these data demonstrate that choice of BME does not induce consistent shifts in dose-response curves for PDOs treated with standard-of-care chemotherapies or trametinib, suggesting that either Matrigel, Cultrex, or UltiMatrix would be appropriate for PDO pharmacotyping analyses.

### BME does not induce significant alterations in dose-response parameters.

We next compared dose-response parameters to gain further insight into subtle changes in dose-response curves both within individual experiments and across all experimental replicates. Within each individual experimental replicate, we performed statistical analyses to identify differences in the half-maximum inhibitory concentration (IC_50_), Hill slope, AUC, and SD of the residuals (Sy.x) between BME lots and sources (22).

**Figure 2:**
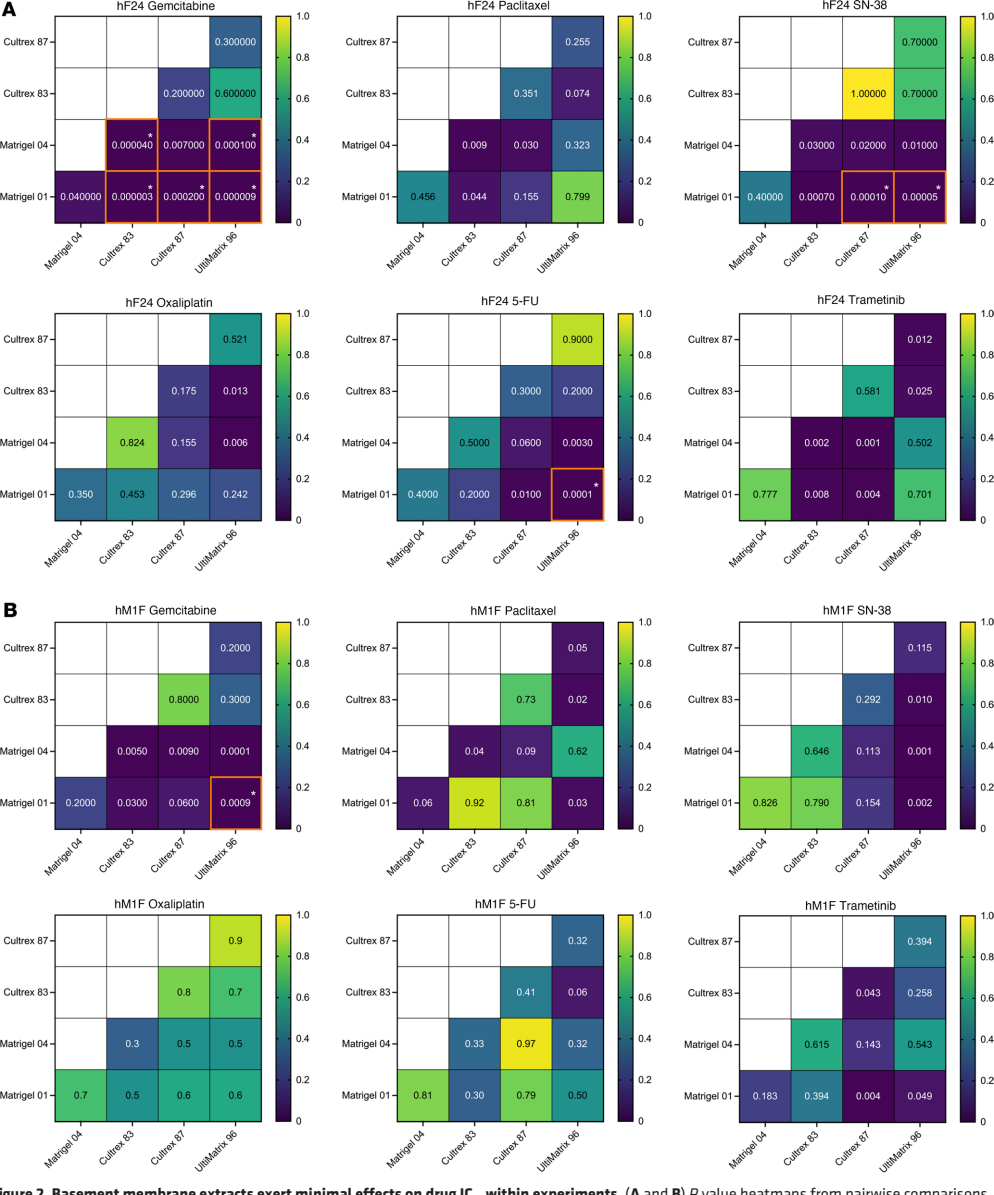
Basement membrane extracts exert minimal effects on drug IC_50_ within experiments. (**A** and **B**) *P* value heatmaps from pairwise comparisons of IC_50_ between BMEs for hF24 (**A**) and hM1F (**B**). Each square represents results of pairwise *t* tests. After Bonferroni adjustment, *t* tests were considered significant if *P* < 0.00027778. Statistically significant comparisons are denoted by an asterisk and orange border.

**Figure 3:**
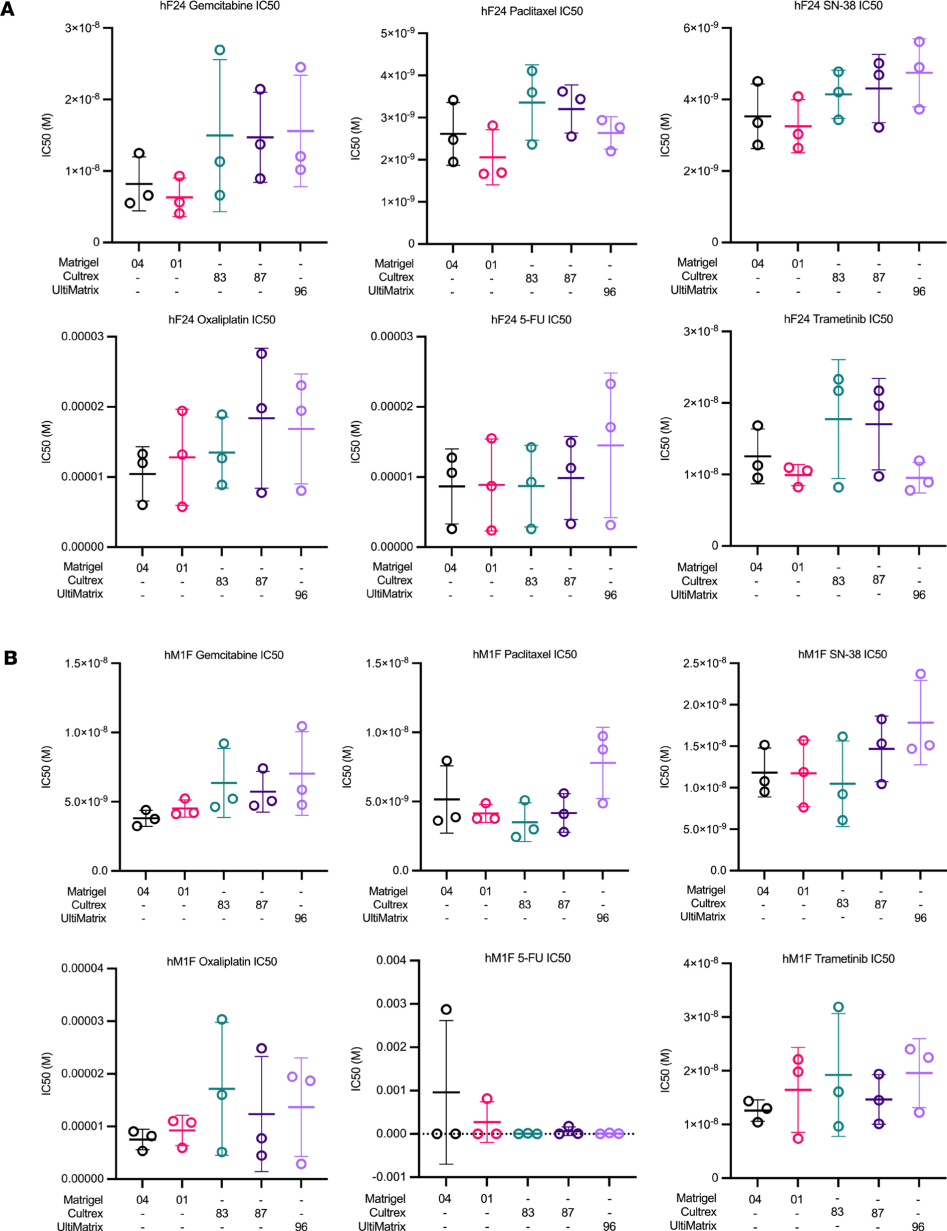
Basement membrane extracts exert minimal effects on drug IC_50_ across experimental replicates. (**A** and **B**) Gemcitabine, paclitaxel, SN-38, oxaliplatin, 5-FU, and trametinib IC_50_ values (M) for hF24 (**A**) and hM1F (**B**) across different BMEs. Data represent mean ± SD of 3 experimental replicates.

We observed a modest number of instances in which IC_50_ values were statistically significant between BMEs within experimental replicates (Figure 2, A and B, and Supplemental Figure 5). Significant pairwise comparisons in IC_50_ were predominantly observed between Matrigel and Cultrex or between Matrigel and UltiMatrix. Across all organoids, drug treatments, and BME comparisons, statistical differences between lots of Matrigel (04 versus 01) were observed only once in a single experimental replicate (Paclitaxel, hF24) (Supplemental Figure 5A). Similarly, statistical differences between lots of Cultrex (83 versus 87) were observed in only 2 experimental replicates (SN-38, hM1F; 5-FU, mT69A) (Supplemental Figure 5, B and D). These data indicate that the lot within the same BME source has little effect on drug IC_50_. Differences in IC_50_ values for oxaliplatin and trametinib were rarely significant, occurring in 1 of 16 experiments (hT1, Matrigel 04 versus Cultrex 87) (Supplemental Figure 5C) and 2 of 16 experiments (hM1F, Matrigel 01 versus UltiMatrix; mT69A, Matrigel 01 versus UltiMatrix, Matrigel 01 versus Cultrex 87, Cultrex 83 versus UltiMatrix) (Supplemental Figure 5, B and D), respectively. Conversely, we consistently observed statistical differences in gemcitabine IC_50_ between Matrigel and Cultrex/UltiMatrix in each experimental replicate of hF24 and hM1F (Figure 2 and Supplemental Figure 5, A and B), as well as in hT1 (*n* = 1 of 2), mT69A (*n* = 1 of 4), and mT69B (*n* = 2 of 4) (Supplemental Figure 5, C–E). Statistical differences in paclitaxel IC_50_ were observed in hF24 (*n* = 1 of 3), hM1F (*n* = 1 of 3), hT1 (*n* = 2 of 2), and mT69B (*n* = 1 of 4) (Supplemental Figure 5, A–C and E). Lastly, differences in 5-FU IC_50_ were observed in only 1 experimental replicate for hF24, mT69A, and mT69B (Figure 2A and Supplemental Figure 5, D and E). To further compare differences in dose response across experimental repeats, we calculated average IC_50_ concentrations (Figure 3 and Supplemental Figure 7). For all hPDOs, we observed no statistically significant alterations in mean IC_50_ values over experimental repeats calculated for any drug as a function of BME (Figure 3 and Supplemental Figure 7A). Similarly, mouse organoids exhibited no statistically significant shifts in drug IC_50_, regardless of which BME that organoids were plated in (Supplemental Figure 7, B and C).

Hill slope is another metric of drug potency that has been suggested to offer higher predictive power than IC_50_ (23). Pairwise comparisons of Hill slope revealed little to no significant differences among BMEs on a per-drug or per-organoid basis (Supplemental Figure 6). The only instances in which Hill slopes were statistically distinct between BMEs were in mouse organoids treated with 5-FU, likely due to the resistant nature of these organoids to 5-FU yielding disparate dose-response curves (Supplemental Figure 6, D and E).

**Figure 4:**
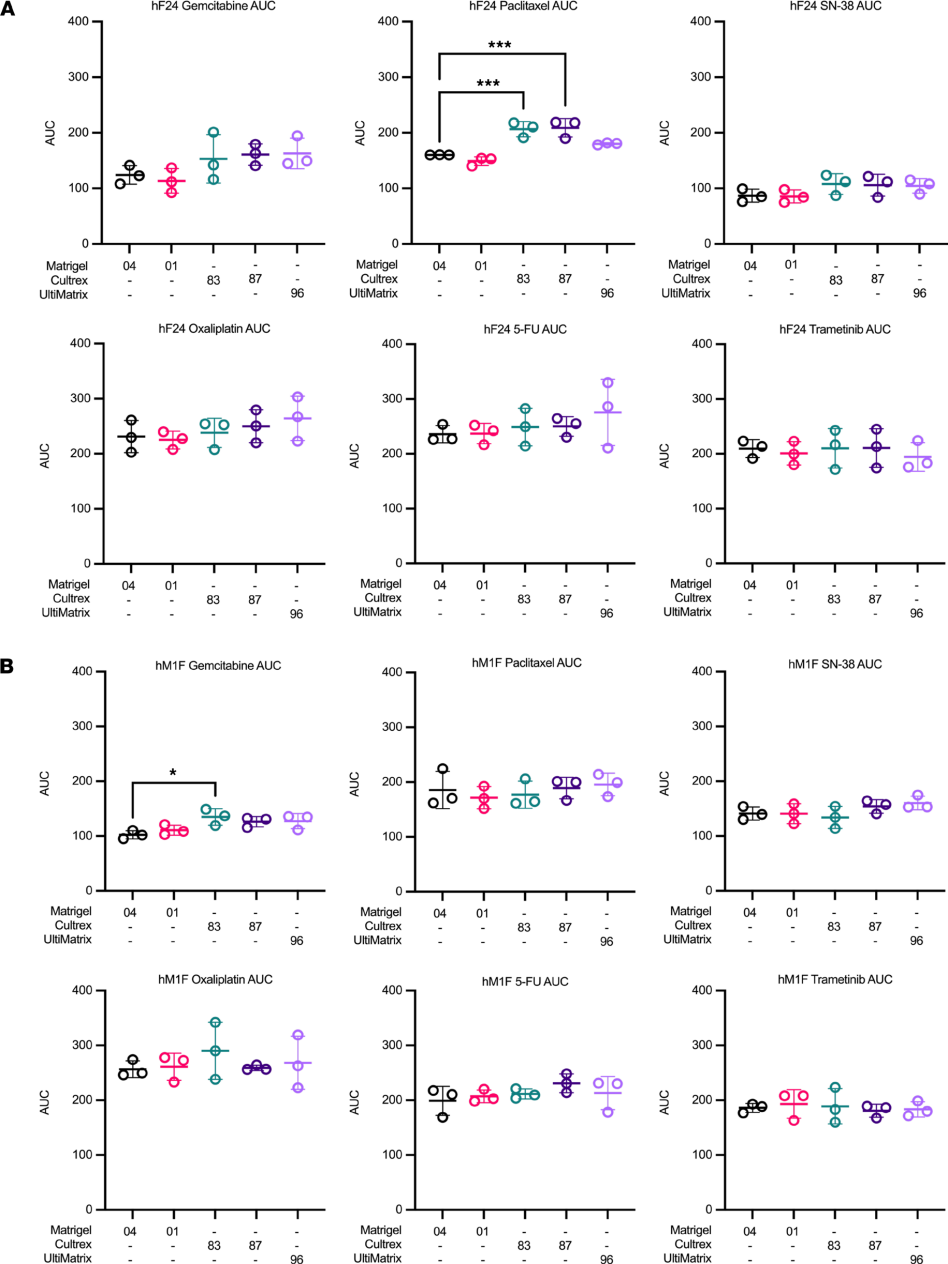
Basement membrane extracts exert minimal effects on AUC. (**A** and **B**) Gemcitabine, paclitaxel, SN-38, oxaliplatin, 5-FU, and trametinib AUC values for hF24 (**A**) and hM1F (**B**) across the different BMEs. Data represent mean ± SD of 3 experimental replicates. Statistical significance was determined by 1-way ANOVA. *P ≤ 0.05, ***P ≤ 0.001 versus Matrigel 04.

We also rarely observed changes in average dose-response curve AUCs across BMEs. Between BME lots, there were no significant AUC differences. AUCs for hF24 treated with paclitaxel were slightly (1.28- to 1.3-fold) but significantly higher in Cultrex 83 or Cultrex 87 compared with Matrigel 04 (Figure 4A). Similarly, calculated AUCs for hM1F treated with gemcitabine were 1.3-fold higher on average in Cultrex 83 compared with Matrigel 04 (Figure 4B). Lastly, AUCs for mT69A treated with paclitaxel were increased in UltiMatrix 96 compared with Matrigel 04 (1.28-fold) (Supplemental Figure 8B). In all other mouse and human drug-organoid combinations tested, there were no significant differences in mean AUC across multiple experiments. These data suggest that, while minimal differences in drug response exist, the most rigorous approach is to complete pharmacotyping analyses using the same commercial BME source.

**Figure 5:**
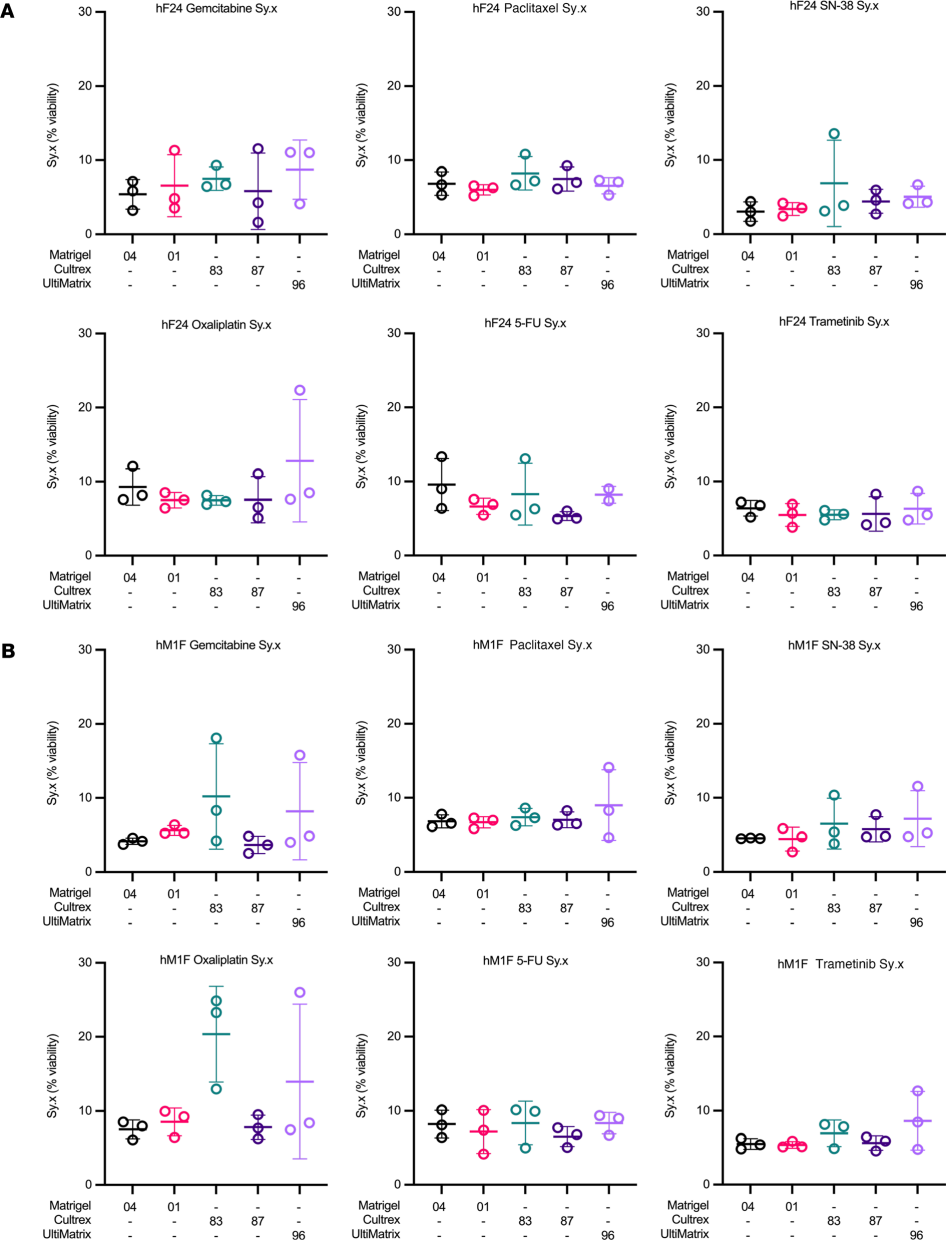
Patient-derived organoid dose-response curve goodness of fit is not significantly altered by basement membrane extract. (**A** and **B**) SD of the residuals (Sy.x) for hF24 (**A**) and hM1F (**B**) treated with gemcitabine, paclitaxel, SN-38, oxaliplatin, 5-FU, or trametinib across the different BMEs. Data represent mean ± SD of 3 experimental replicates.

We next sought to analyze dose-response curve goodness of fit as a parameter of plating consistency in each BME within and across experimental replicates. We measured the Sy.x as an estimate of goodness of fit. In general, higher Sy.x values indicate larger deviations of data points from the fit regression curve. Sy.x values for Cultrex 83 were relatively consistent across experimental replicates, with low absolute values (<10%) and low interexperimental variability (Figure 5). The largest variations in Sy.x across experimental replicates in Cultrex were hF24 treated with SN-38 and 5-FU, and hM1F treated with gemcitabine and oxaliplatin (Figure 5). Sy.x values were larger on average for Cultrex 83 in hM1F treated with oxaliplatin (mean = 20.37%). Cultrex 87 performed consistently as well (ranging from 1.6% to 11.5%), with the most statistical variance across experiments observed in hF24 treated with gemcitabine (Figure 5A). UltiMatrix 96, relative to the other BMEs, exhibited higher experimental variability in Sy.x values, with greater variance observed in hF24 treated with gemcitabine (4.1%–11%) and oxaliplatin (7.6%–22.3%) and in hM1F treated with gemcitabine (4.0%–15.8%), paclitaxel (4.6%–14.1%), oxaliplatin (7.5%–26%), and trametinib (4.8%–12.6%) (Figure 5). Matrigel 04 yielded consistent and relatively low Sy.x values (<10%) across all experimental replicates, with the exception of hF24 treated with 5-FU (6.3%–13.3%) (Figure 5A). Similarly, Matrigel 01 demonstrated low and consistent Sy.x values across treatments — experimental replicates — and hPDOs tested, as evidenced by low SDs and low Sy.x absolute values (Figure 5 and Supplemental Figure 9). Overall, these results indicate better dose-response curve goodness of fit from experiment to experiment when human organoids are cultured in Matrigel.

**Figure 6:**
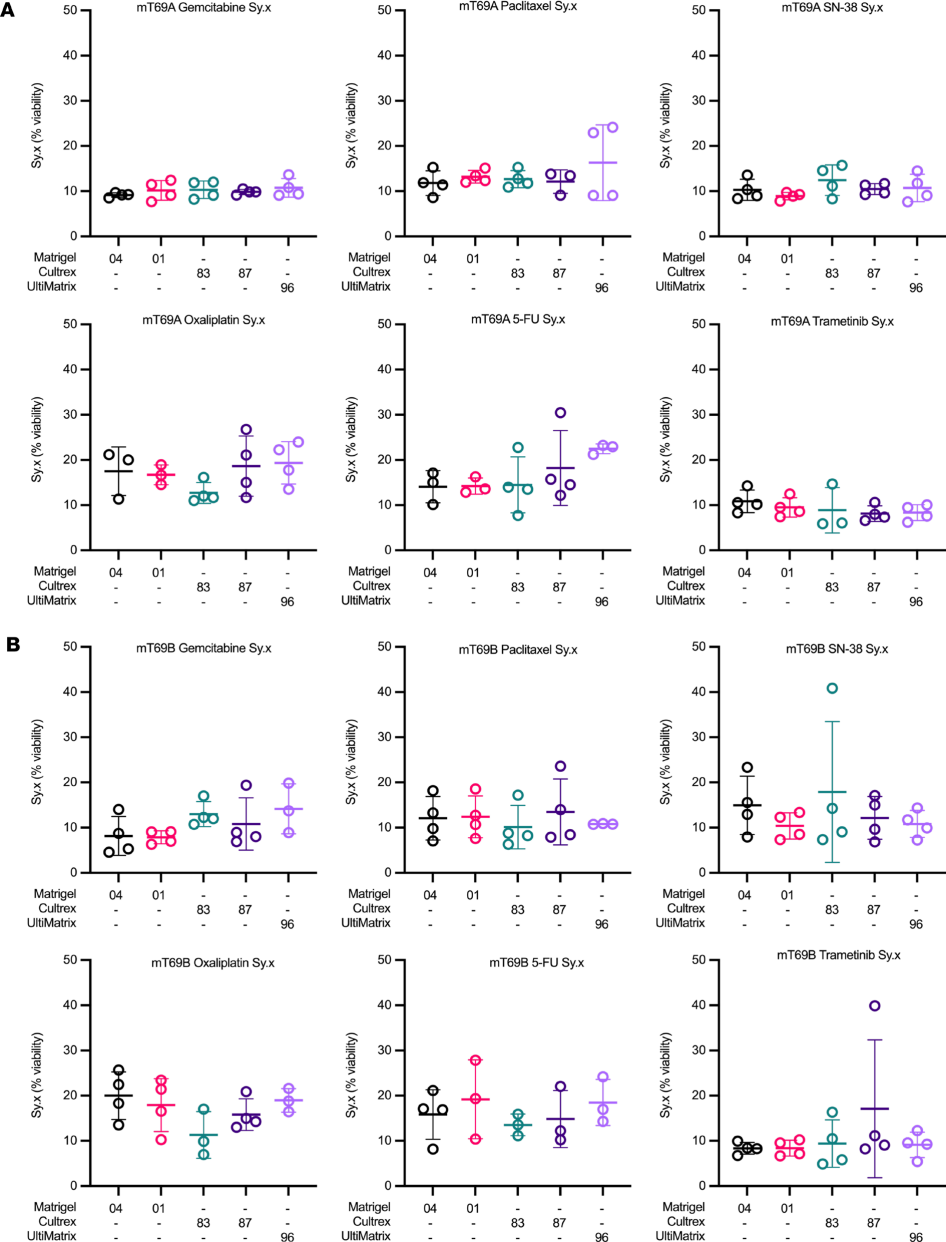
Mouse organoid dose-response curve goodness of fit is not significantly altered by basement membrane extract. (**A** and **B**) SD of the residuals (Sy.x) for mT69A (**A**) and mT69B (**B**) treated with gemcitabine, paclitaxel, SN-38, oxaliplatin, 5-fluorouracil (5-FU), or trametinib across the different BMEs. Data represent mean ± SD of 4 experimental replicates. Outliers were determined using Grubbs’ test (α = 0.05).

For mouse organoids, similar trends were observed, albeit with higher absolute Sy.x values across almost all conditions (4.5%–40.8%) (Figure 6). In general, mouse organoids were more sensitive to single-cell dissociation protocols and had higher growth rates, both of which contributed to increased variance and necessitate shorter pharmacotyping experimental time courses (3 days for mouse organoids versus 5 days for hPDOs). Over the course of 4 experimental replicates, UltiMatrix 96 demonstrated a high degree of statistical variability in Sy.x values relative to the other BMEs tested, notably for mT69A treated with paclitaxel (9.0%–24.1%) and oxaliplatin (13.5%–23.9%) and for mT69B treated with gemcitabine (8.81%–19.8%) (Figure 6). Relative to the other BMEs tested, Cultrex lots also exhibited high levels of variability in Sy.x. Cultrex 83 exhibited a high degree of variability for mT69A treated with 5-FU (7.7%–22.7%) and trametinib (5.9%–14.7%) and for mT69B treated with paclitaxel (6.3%–17.1%) and SN-38 (7.3%–40.8%) (Figure 6). Similar variability in Sy.x across experimental replicates was observed for Cultrex 87 in mT69A treated with oxaliplatin (11.7%–26.8%) and 5-FU (12.2%–30.5%) and in mT69B treated with gemcitabine (6.9%–19.4%), paclitaxel (7.9%–23.6%), and trametinib (8.2 %–39.9%) (Figure 6). Sy.x values were consistent for mT69B treated with paclitaxel when cultured in UltiMatrix 96 (10.7%–10.9%) (Figure 6B). Both Matrigel 04 and Matrigel 01 showed relatively consistent Sy.x values in the mouse organoid context but exhibited some biological and experimental variance. Most notably, mT69A and mT69B were relatively resistant to oxaliplatin and 5-FU and exhibited relatively high Sy.x absolute values (20%–30%) in these conditions compared other drug treatments (Figure 6).

Overall, we observed no statistically significant differences in average Sy.x values for either human or mouse organoids across BMEs and all drugs (Figure 6 and Supplemental Figure 9). However, for human and mouse organoids, both lots of Matrigel exhibited more consistent Sy.x values across experimental replicates compared with Cultrex or UltiMatrix, as evidenced by lower variance in Sy.x values across experiments (Figure 6). Lot-to-lot variability was more pronounced for Cultrex compared with Matrigel for both human and mouse organoids. Collectively, these data indicate that drug dose-response curve fitting was stable across the BMEs tested.

### Gene expression and organoid subtype classification are not influenced by BME.

**Figure 7:**
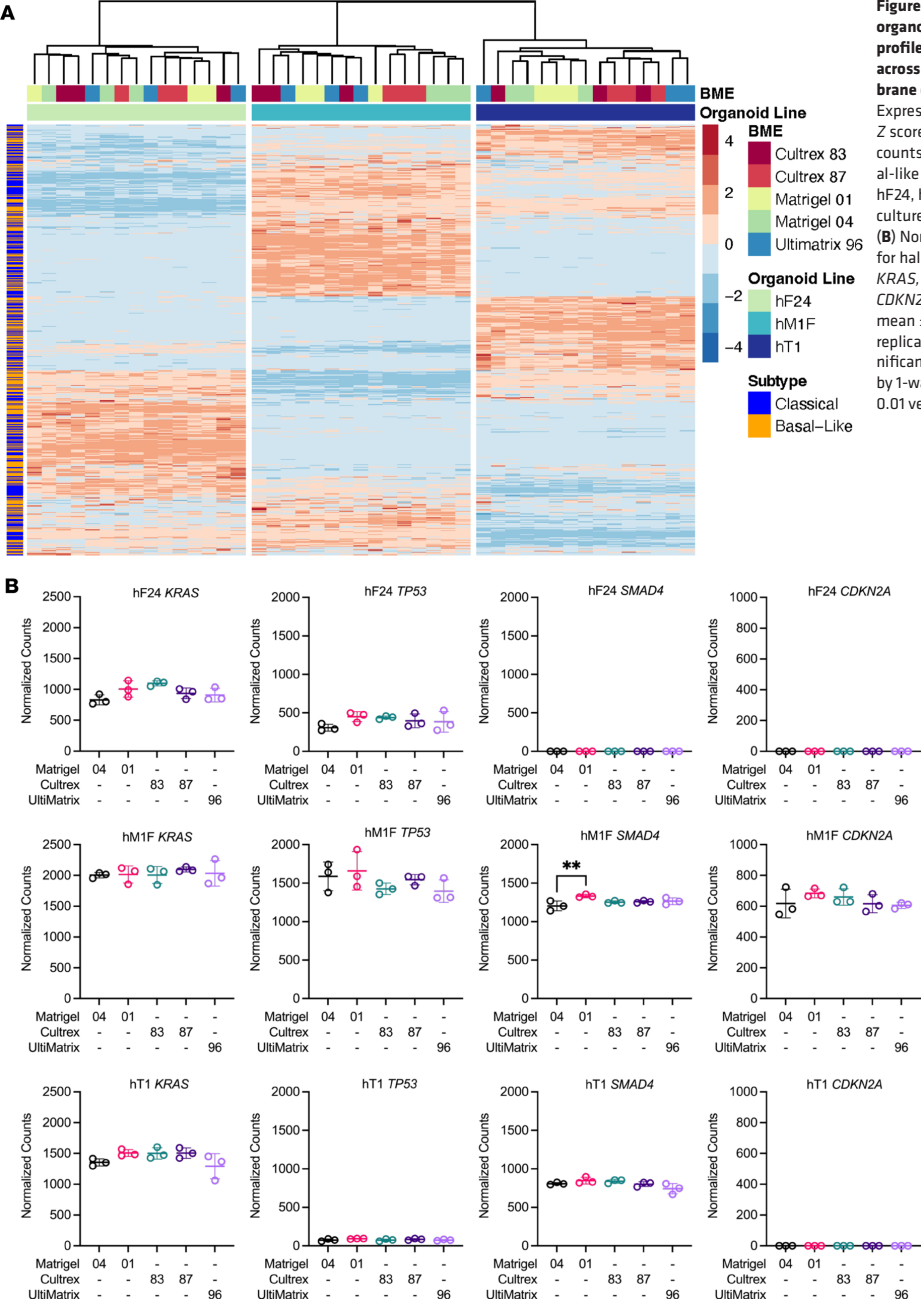
Patient-derived organoid gene expression profiles are consistent across basement membrane extract types. (**A**) Expression heatmap of *Z* scores of normalized counts of classical or basal-like hallmark genes in hF24, hM1F, and hT1 when cultured in different BMEs. (**B**) Normalized counts for hallmark PDA genes *KRAS, TP53, SMAD4,* and *CDKN2A*. Data represent mean ± SD of 3 technical replicates. Statistical significance was determined by 1-way ANOVA. **P ≤ 0.01 versus Matrigel 04.

Although we did not observe substantial shifts in drug response as a function of BME lot or source, alterations in intracellular signaling and cell state represented another potential variable influenced by BME composition. Therefore, we also investigated BME-induced changes in gene expression or PDA subtype classification (24). To this end, mouse organoids (mT69A, mT69B, mT9) and human PDOs (hF24, hM1F, hT1) were cultured in each BME (Matrigel 04, Matrigel 01, Cultrex 83, Cultrex 87, and UltiMatrix 96) for 3 days and subjected to RNA-Seq analyses (Supplemental Figure 1B). Multivariate analysis of resultant *Z* scores of organoid normalized counts indicated that both mouse and human samples clustered well based on organoid line (Figure 7A and Supplemental Figure 10, C and D). However, samples did not cluster markedly based on BME (Figure 7A and Supplemental Figure 10, C–F). Gene expression was largely unchanged between Matrigel lots in both human and mouse organoids (0–2 differentially expressed genes, 20 in hM1F) (Supplemental Figure 10, A and B). Similarly, minimal gene expression changes were observed in human and mouse organoids between Cultrex lots (0–4 differentially expressed genes) (Supplemental Figure 10, A and B). Between BME sources, we observed more changes in gene expression (absolute log_2_ fold change [abs(lfc)] > 1, FDR ≤ 0.05), but these were not consistent between organoid lines. For example, hT1 (Stage 2B) displayed 522 differentially expressed genes between Matrigel 04 and UltiMatrix, whereas hF24 (Stage 4) and hM1F (Stage 4, lung metastasis) presented 0 (Supplemental Figure 10A). Similarly, mT69B (KPC PDA primary tumor) displayed 90 differentially expressed genes between Matrigel 04 and Cultrex 87, whereas mT69A (KPC PDA metastasis) presented with 13 (Supplemental Figure 10B). These results indicate that BME does not substantially alter PDO gene expression profiles.

To investigate pathways that could explain differences observed in organoid proliferation, differential expression was calculated between BMEs with significantly decreased proliferation compared with Matrigel. Gene set enrichment analysis (GSEA) was performed on the Reactome database for each organoid. We observed a trend toward upregulation of terms related to cell cycle and translation in hF24, hM1F, and mT69A (Supplemental Figure 11A). Normalized counts of top candidate genes related to proliferation revealed upregulation of a number of genes when hPDOs were cultured in Matrigel, such as *ANXA6*, elevated levels of which have been described in PDA (25); *AQP5*, implicated in PDA cell proliferation and biophysical properties of cell membranes (26); and *PIK3AP1*, a protein capable of activating AKT phosphorylation in gastric cancer (27) (Supplemental Figure 11B). Similarly, in Matrigel, mouse organoids exhibited enriched expression of *Spp1*, a gene previously identified as a marker of undifferentiated pancreatic progenitors in the ductal niche (28, 29) (Supplemental Figure 11C). Across both species, only *RDH10* was downregulated by Matrigel in both mouse and human organoids, with the trend broken by UltiMatrix in hT1 (Supplemental Figure 11, D and E). Interestingly, RDH10 has been demonstrated to be indispensable for the synthesis of retinoic acid required for the recruitment and differentiation of early pancreas progenitors (30).

Previous work defined molecular subtypes of PDA. Patients with basal-like PDA exhibit poor prognoses and increased therapeutic resistance compared with the classical subtype (24). Changes to molecular subtype would have major implications on the predictive power of PDOs in precision medicine approaches. To determine whether BME influenced hPDO subtype classification, we delineated significant changes to enrichment of basal-like and classical differentially expressed genes (Supplemental Data). Pairwise comparisons of subtype gene enrichment indicated no significant difference in basal-like or classical gene enrichment between Matrigel 04 and Matrigel 01 or between Cultrex 83 and Cultrex 87 (Supplemental Figure 12). A number of comparisons were enriched for both classical and basal-like gene sets (Supplemental Figure 12). For instance, hM1F displayed upregulation of both basal-like and classical hallmark genes when cultured in Cultrex 83, Cultrex 87, or UltiMatrix (Supplemental Figure 12). Analysis of individual genes implicated in the etiology of PDA revealed that BME did not significantly alter hPDO expression levels of *KRAS*, *TP53*, or *CDKN2A*. A slight but statistically significant increase in *SMAD4* expression was detected in hM1F cultured in Matrigel 01 compared with Matrigel 04 (Figure 7B). Similarly, we observed slight yet statistically significant increases in *ERBB3* in all 3 hPDOs cultured in Cultrex or UltiMatrix (Supplemental Figure 13). Expression of *EGFR* was increased in hT1 cultured in Cultrex 87 relative to Matrigel 04 (Supplemental Figure 13). However, we did not observe any significant changes in expression levels of *MYC*, *GATA6*, *MAP2K1*, *MAP2K2*, *SOX9*, *RB1*, *BRCA2*, or *ERBB2* or in genes related to drug resistance such as *HNF1A* and *ABCB1* (Supplemental Figure 13). Overall, these data indicate that the potential influence of BME on organoid gene expression, cell state, and subsequent response to targeted therapies must be carefully considered when developing protocols for organoid-guided therapy studies.

## Discussion

The ability of PDOs to mirror patient genetics, transcriptomics, and drug response in retrospective studies prompted the onset of several clinical trials to test the benefit of using PDOs to inform treatment decisions. However, previous studies demonstrated the plasticity of PDOs in response to varying culture conditions and potential effect on drug response (16, 17). With multiinstitutional clinical trials underway, the need to investigate factors that affect the robustness of PDO genetics, transcriptomics, and drug testing is imperative. BME are central to most PDO efforts. Previous studies demonstrate variability in protein composition, presence of xenogenic bioactive compounds, and scaffold stiffness between batches. Despite this variability, commercially available BMEs remain critical and prevalent in organoid efforts. Given the multiyear nature of PDO-related clinical trials, more than 1 lot of BME will be required for the duration of these studies. In addition, limitations in availability due to supply chain issues has necessitated that some efforts switch between alternate commercial sources. However, the influence of BME source and lot on organoid response to drug treatment was unknown.

To investigate the extent to which BME affects organoid growth, drug response, and transcriptomics, we compared various commercially available BMEs on previously established mouse and human PDA organoids. We consistently observed a decrease in mouse and human organoid growth when cultured in Cultrex or UltiMatrix compared with Matrigel. While this could arise from a lack of an adaptation period, multiple passages of mouse organoids in Cultrex does not rescue their growth rate. Despite this growth deficit, we observed no substantial shifts in drug response or global gene expression as a function of BME source or lot. The generation efficiency of PDA organoids in Matrigel is 75% (8). This study made use of previously established organoids. Given the effect of BME source on PDO growth, it will be important to delineate the effect of BME on isolation efficiency in future work. For established organoids, Matrigel, Cultrex, or UltiMatrix BMEs are all viable options for pharmacotyping experiments. Altogether, this work demonstrates that precision medicine approaches using organoids are robust and that ongoing clinical trials are unlikely to be affected by different lots or commercial sources of BME.

## Methods

### Human specimens, organoids, and cell culture conditions.

Organoids used in this study were generated at Cold Spring Harbor Laboratory (Cold Spring Harbor, New York, USA) (7, 8, 31). Patient-derived and mouse organoids were cultured as previously described (7, 8). For organoid generation efficiency studies, material from dissociated tissue specimens were plated in equal densities in Matrigel 01, Matrigel 04, Cultrex 83, Cultrex 87, or UltiMatrix 96 and overlaid with organoid feeding media. In short, mouse organoid media contained Advanced DMEM/F12, HEPES, glutamax, B27 supplement, and penicillin/streptomycin (Thermo Fisher Scientific); *N*-acetylcysteine and nicotinamide (Sigma-Aldrich); EGF, FGF-10, gastrin, and A83-01 (R&D); and Noggin and R-spondin 1 (Qkine). For human organoid culture, Wnt3a was also included in the media. Organoid nomenclature is defined as: hT, human tumor obtained from resection; hF, human fine-needle biopsy obtained by fine-needle aspiration or core biopsy; hM, human metastasis obtained from direct resection of metastases following rapid autopsy or VATS resection; mT, murine tumor. All organoid models were routinely tested for *Mycoplasma* at Salk Institute. Additional hPDO and mouse organoid characteristics are available in Supplemental Tables 1 and 2.

### Mouse specimens.

All mouse experiments were performed in accordance with procedures approved by the IACUC at Salk Institute for Biological Studies. Mice harboring the *LSL-Kras^G12D^*, *LSL-Trp53^R172H^*, and *Pdx-1-Cre* alleles were identified by sending tissue samples to Transnetyx for genotyping. Three mice (A5462, female, 8 weeks old; A5504, female, 8 weeks old; A5505, male, 9 weeks old) were euthanized and necropsied, with the pancreas processed for histology and organoid generation as described above. Mouse pancreas histology was assessed using H&E staining.

### BME.

Both human and mouse organoids were maintained in Corning Matrigel lot no. 1062004 during the growth and expansion phase. For pharmacotyping comparisons, organoids were plated in either Corning Matrigel (growth factor reduced, phenol red free) lot no. 1062004 (Matrigel 04), Corning Matrigel (growth factor reduced, phenol red free) lot no. 0287001 (Matrigel 01), RnD Cultrex reduced growth factor BME lot no. 1564183 (Cultrex 83), RnD Cultrex reduced growth factor BME lot no. 1586187 (Cultrex 87), or Cultrex UltiMatrix reduced growth factor BME lot no. 1637796 (UltiMatrix 96).

### Pharmacotyping of organoids.

Organoids were dissociated into single cells using a diluted solution of TrypLE Express (Thermo Fisher Scientific). Equal numbers of single cells (1,500 viable cells per well) were plated in a 20 μL suspension of 10% BME in ultra-low attachment 384-well plates (Corning). Twenty-four hours after plating, reformation of organoids was visually verified, and therapeutic compounds were applied using a digital dispenser (Tecan). Chemotherapies were tested in triplicate 10-point dose-response curves. Gemcitabine, paclitaxel, SN-38 (active metabolite of irinotecan), and trametinib ranged from 0.5 nM to 5μM; oxaliplatin and 5-fluorouracil ranged from 50 nM to 100 μM. Compounds were dissolved in DMSO, and all treatments were normalized to 0.5% DMSO content. Mouse organoids underwent treatment for 3 days, while human organoids underwent treatment for 5 days. After treatment, cell viability was assessed using Cell Titer Glo (Promega) per manufacturer’s instructions on a Tecan Spark Cyto plate reader. A 4-parameter log-logistic (LL4) function with upper limit equal to the mean of the lowest dose values was fit to the data (viability versus dose) with Graphpad Prism 9. The IC_50_ values, AUC, and Sy.x for each dose-response curve were calculated using Graphpad Prism 9. Each pharmacotyping experiment was carried out at least 2 times.

### RNA isolation.

For RNA-Seq comparisons, organoids were maintained in Corning Matrigel lot no. 1062004 (Matrigel 04) and then plated in either Corning Matrigel (growth factor reduced, phenol red free) lot no. 1062004 (Matrigel 04), Corning Matrigel (growth factor reduced, phenol red free) lot no. 0287001 (Matrigel 01), RnD Cultrex reduced growth factor BME lot no. 1564183 (Cultrex 83), RnD Cultrex reduced growth factor BME lot no. 1586187 (Cultrex 87), or Cultrex UltiMatrix reduced growth factor BME lot no. 1637796 (UltiMatrix 96). Each BME condition was plated in triplicate, and organoids were grown for 3 days before harvesting in TRIzol (Invitrogen) and snap frozen. RNA was isolated using the RNeasy Mini Kit (Qiagen). RNA quality control was performed for all samples using a Qubit Fluorometer (Invitrogen) and TapeStation (RIN greater than 8.5) (Agilent) before RNA-Seq analyses.

### RNA-Seq analyses.

Library construction was conducted using the TruSeq RNA library prep kit (Illumina) with 500 ng RNA input. Raw reads were trimmed with Trim Galore v0.4.4_dev (https://www.bioinformatics.babraham.ac.uk/projects/trim_galore/) and quality checked with FastQC v0.11.8 (http://www.bioinformatics.babraham.ac.uk/projects/fastqc). Trimmed reads were then aligned to the hg38 human reference genome with STAR aligner v2.5.3a (32), and converted to gene counts with HOMER’s analyzeRepeats.pl script (33).

### Differential gene expression analysis.

Gene counts where normalized and queried for differential expression using DESeq2 v1.30.0 (34). For each pairwise comparison, genes with fewer than 10 total raw counts across all samples were discarded prior to normalization, and genes with an absolute log_2_ fold change > 1 and an FDR-corrected *P* ≤ 0.05 were pulled as significant.

### GSEA.

For each organoid, differential expression was calculated between Matrigel and BMEs with significantly decreased proliferation compared with Matrigel (hF24, Cultrex 87 + UltiMatrix versus Matrigel 04 + Matrigel 01; hM1F, Cultrex 87 + UltiMatrix versus Matrigel 04 + Matrigel 01; hT1, Cultrex 87 + Cultrex 83 versus Matrigel 04 + Matrigel 01; mT69A, Cultrex 87 + UltiMatrix versus Matrigel 04; mT69B, Cultrex 83 + Cultrex 87 + UltiMatrix versus Matrigel 04 + Matrigel 01). GSEAs were run on the Reactome database for each organoid using the *P* values of differential expression × sign of log_2_ fold change in WebGestaltR.

### Linear regressions.

Candidate genes were queried by running a linear regression of (linear regression of gene normalized counts versus proliferation fold change) for each organoid. Genes with a significant correlation between expression and proliferation in all organoids were selected as top candidate genes.

### Statistics.

A LL4 function with an upper limit equal to the mean of the lowest dose values was fit to the dose-response data (viability versus dose) with Graphpad Prism 9. The IC_50_ values, AUC, and Sy.x for each dose-response curve were calculated using GraphPad Prism 9. Each pharmacotyping experiment was carried out for *n* = 2–4 experimental replicates. Differences among means were analyzed using 1-way ANOVA with Dunnett’s multiple comparisons test. Outliers were determined using Grubbs’ test (α = 0.05).

To compare estimated dose-response curve parameters across different BMEs within an experimental replicate, 2-tailed *t* tests were conducted on the IC_50_ and Hill slope to test the null hypothesis of zero difference in the parameters among BMEs using R 4.0.3 and package drc (22, 35). The Bonferroni method was used to adjust for multiple comparisons, and a comparison between a pair of BMEs was considered significant if the *P* value was below 0.05 divided by the total number of pairwise comparisons. Consequently, 2-tailed pairwise *t* tests were considered significant if *P* < 0.00027778 (hPDO) or *P* < 0.00041667 (mouse organoids).

To assess the model fitting and compare the log-logistic models with other alternatives, including Weibull models, a sensitivity analysis was conducted. The Akaike information criterion (AIC) was used for the comparison. The LL4 model consistently yielded smaller AIC values, suggesting this model as the most appropriate model-fitting strategy. Analyses presented from the aforementioned *t* tests on IC_50_ and Hill slope were conducted first on data fitted with the LL4 model and then on data fitted with Weibull models when appropriate.

### Study approval.

Organoids used in this study were generated at Cold Spring Harbor Laboratory (7, 8, 31) and generated from human biospecimens collected by the Moores Cancer Center Biorepository from consented patients under a University of California San Diego Human Research Protections Program IRB–approved study (HRPP no. 181755) and following approval from the Salk Institute IRB. Biorepository patients provided a written consent that is maintained in the Biorepository archives. All mouse experiments were performed in accordance with procedures approved by the IACUC at Salk Institute for Biological Studies. 

### Data availability.

Raw data for RNA-Seq are publicly available on NCBI Gene Expression Omnibus (accession no. GSE232170; https://www.ncbi.nlm.nih.gov/geo/query/acc.cgi?acc = GSE232170). Raw data for figures are available in the Supporting Data Values XLS file. Supporting analytic code can be accessed from the corresponding author upon request.

## Author contributions

JCL, SRO, KLP, and SY generated and maintained organoid cultures, acquired materials, and designed and conducted the experiments. IN, AML, and HT coordinated and provided receipt of clinical research specimens. JCL and XL acquired and analyzed the data. KL performed statistical analysis on RNA-Seq data. JZ directed and provided statistical analysis for dose-response curve comparisons. JCL, XL, JZ, and DDE wrote the manuscript. DDE conceived the project and directed the experiments.

## Supplementary Material

Supplemental data

Supplemental data set 1

Supporting data values

## Version 1 12/05/2023

In-Press Preview

## Version 2 01/09/2024

Electronic publication

## Version 3 01/11/2024

An authorship note was added

## Version 4 02/28/2024

Removed extra page from layout
