# Supplementary material for: The effect of extracellular matrix on the precision medicine utility of pancreatic cancer patient–derived organoids
Source: JCI Insight. 2024 Jan 9;9(1):e172419. doi: 10.1172/jci.insight.172419 (PMC10906458; doi:10.1172/jci.insight.172419)
Supplement: Supplemental data [file jciinsight-9-172419-s178.pdf]

Supplemental Table 1

PDO	Origin	Site	Type of sample	Sex	Age	Race	Stage	KRAS (allele frequencies)	TP53 (allele frequencies)	SMAD4 (allele frequencies)	CDKN2A (allele frequencies)	Treatment prior to organoid
hF24*	H. sapiens	Pancreas	EUS FNA	F	57	Caucasian	4	G12V 82%	F113V (100%)	LOSS (N/A)	DEEP LOSS (N/A)	NO
hM1F*	H. sapiens	Lung	RAP	M	55	Caucasian	4	G12D 73%	R175H (100%)	WT (N/A)	WT (N/A)	YES
hT1*	H. sapiens	Pancreas	Resection	M	65	Caucasian	2B	G12V 39%	R213* (100%)	WT (N/A)	DEEP LOSS (N/A)	NO
449**	H. sapiens	Liver	Wedge biopsy	M	73	Caucasian	4	--	WT**	--	--	YES

*H. Tiriac *et al.*, Organoid Profiling Identifies Common Responders to Chemotherapy in Pancreatic Cancer. *Cancer Discov* **8**, 1112-1129 (2018).
**UCSD Moores Cancer Center Biorepository and Tissue Technology Shared Resource.

Supplemental Table 2

PDO	Origin	Sex	Site	Type of sample
mT69A	M. musculus	F	Pancreas	KPC B6, PDAC local pancreatic metastasis
mT69B	M. musculus	F	Pancreas	KPC B6, PDAC primary tumor
mT9	M. musculus	F	Pancreas	KPC PDAC primary tumor
A5462	M. musculus	F	Pancreas	KPC B6, pancreas harboring premalignant lesions
A5504	M. musculus	M	Pancreas	KPC B6, pancreas harboring premalignant
A5505	M. musculus	M	Pancreas	KPC B6, pancreatic tumor with mixed adenocarcinoma and sarcomatoid histopathology

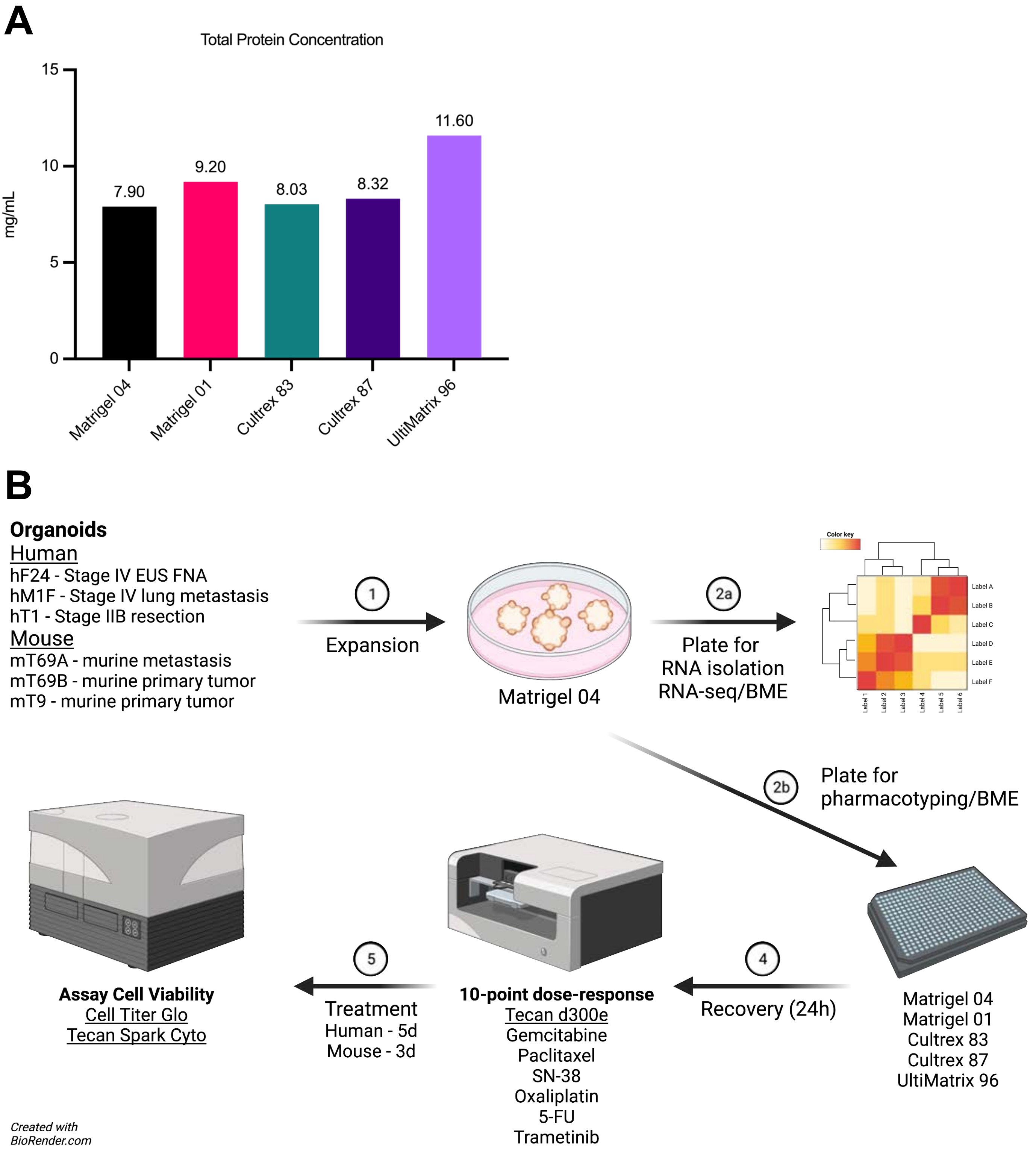

Supplemental Figure 1

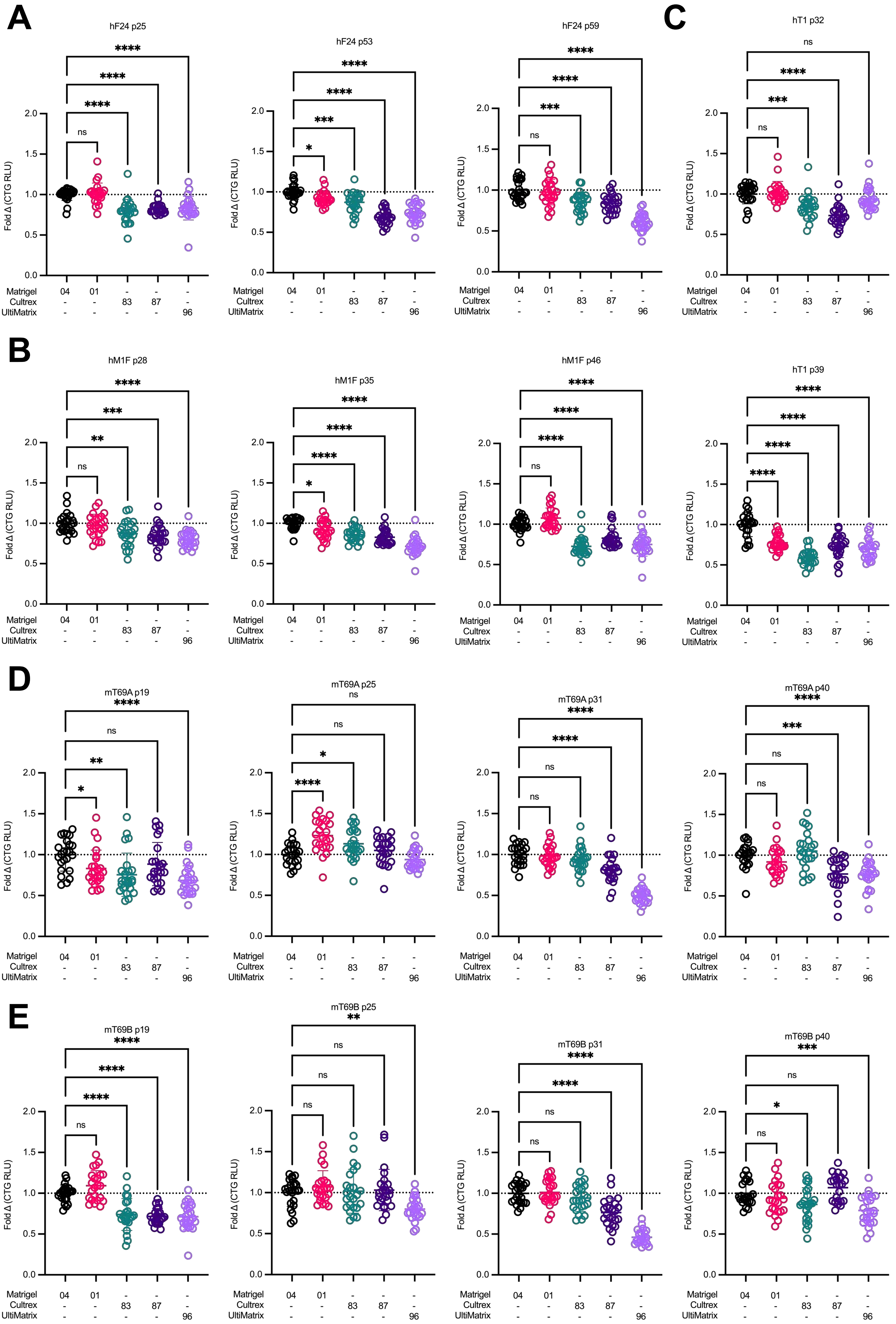

Supplemental Figure 2

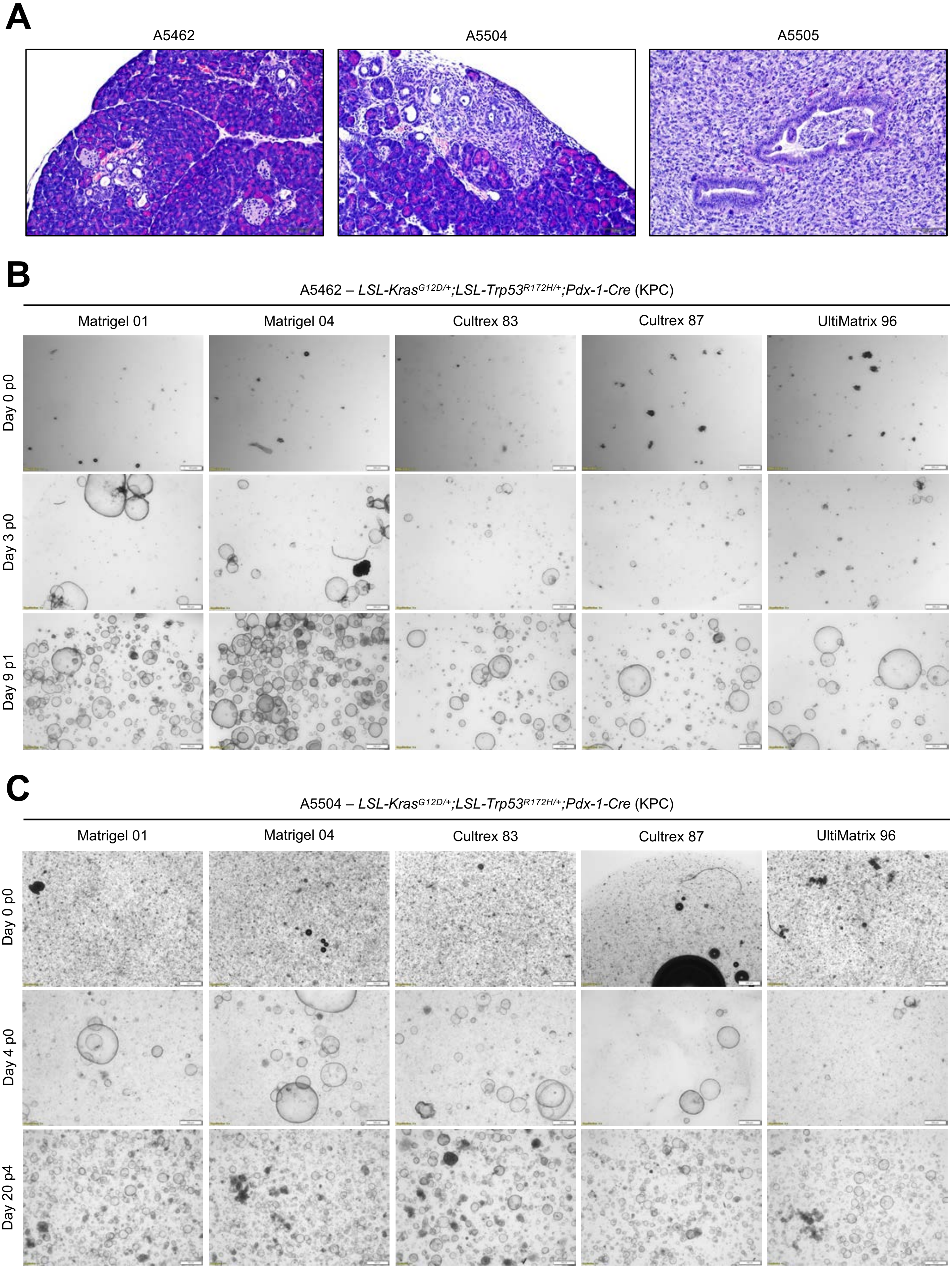

Supplemental Figure 3A-C

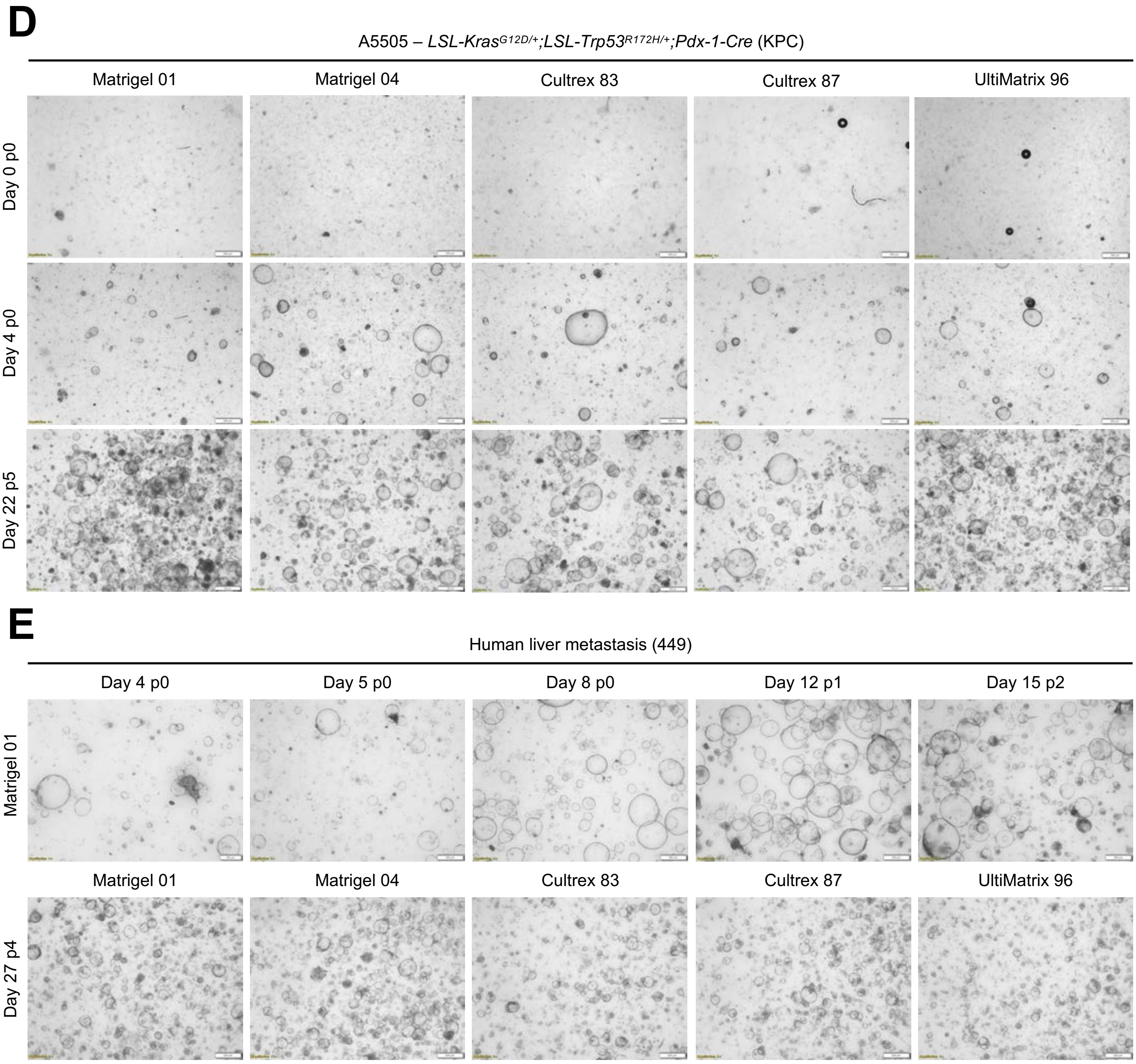

Supplemental Figure 3D-E

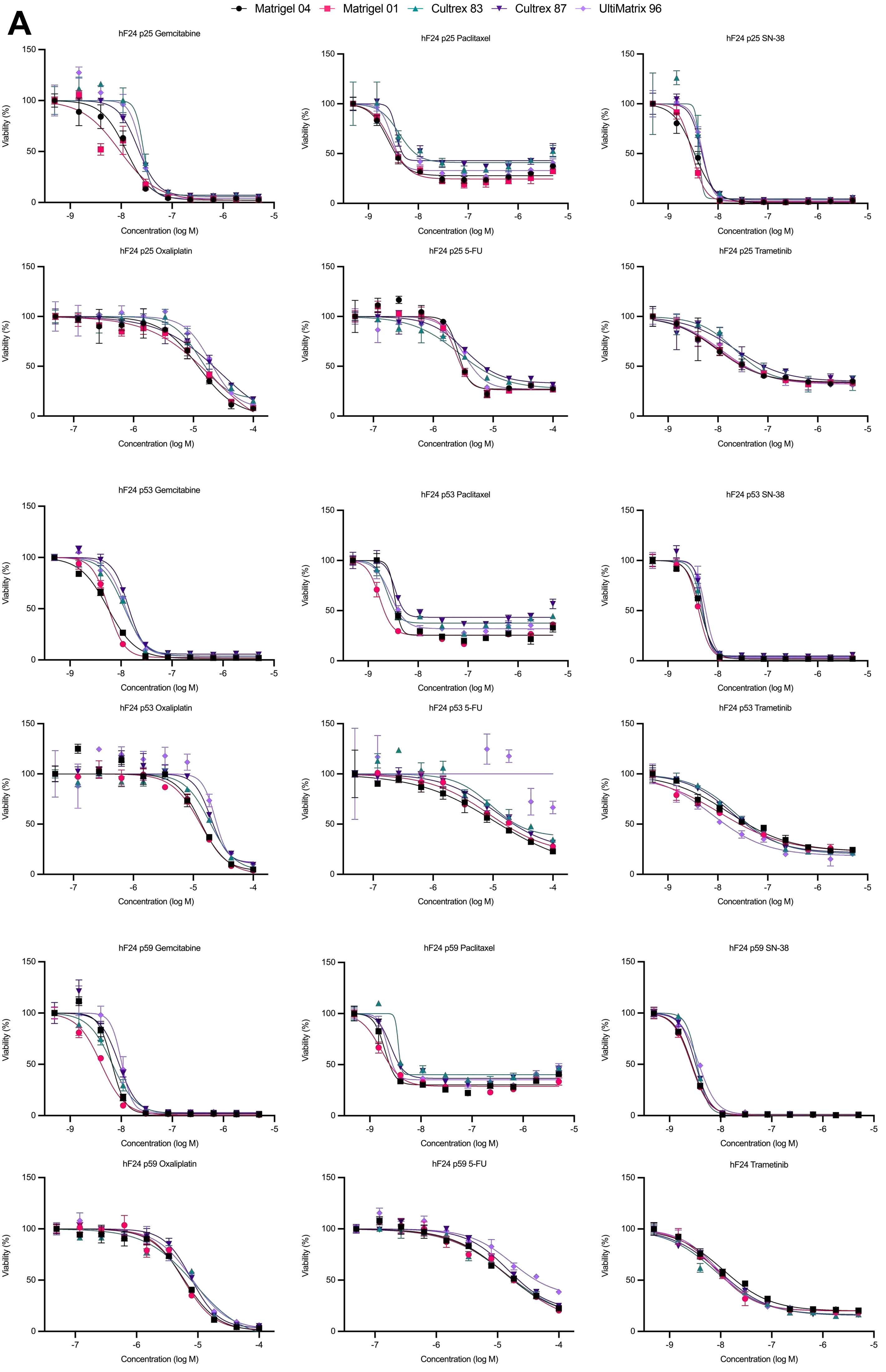

Supplemental Figure 4A

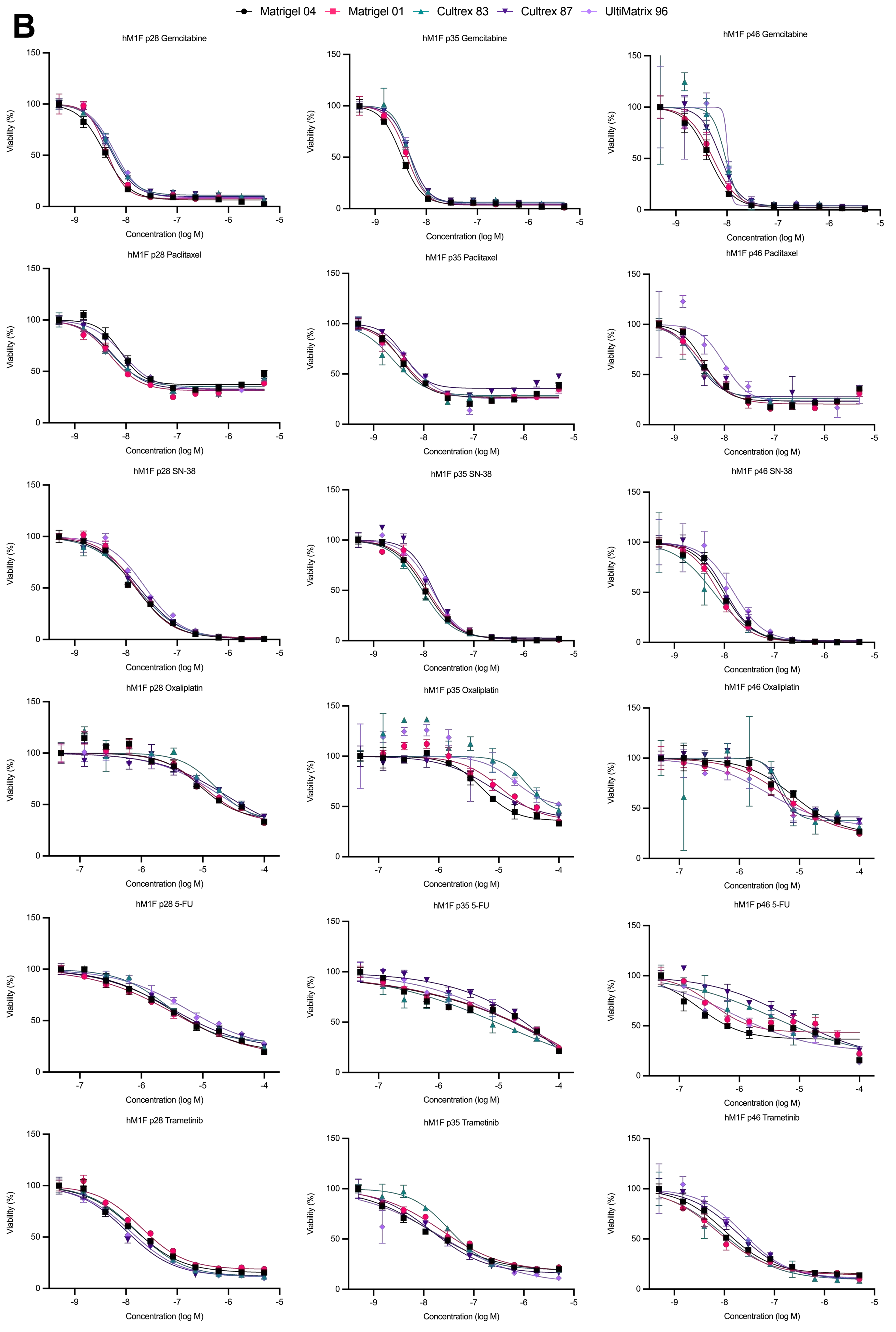

Supplemental Figure 4B

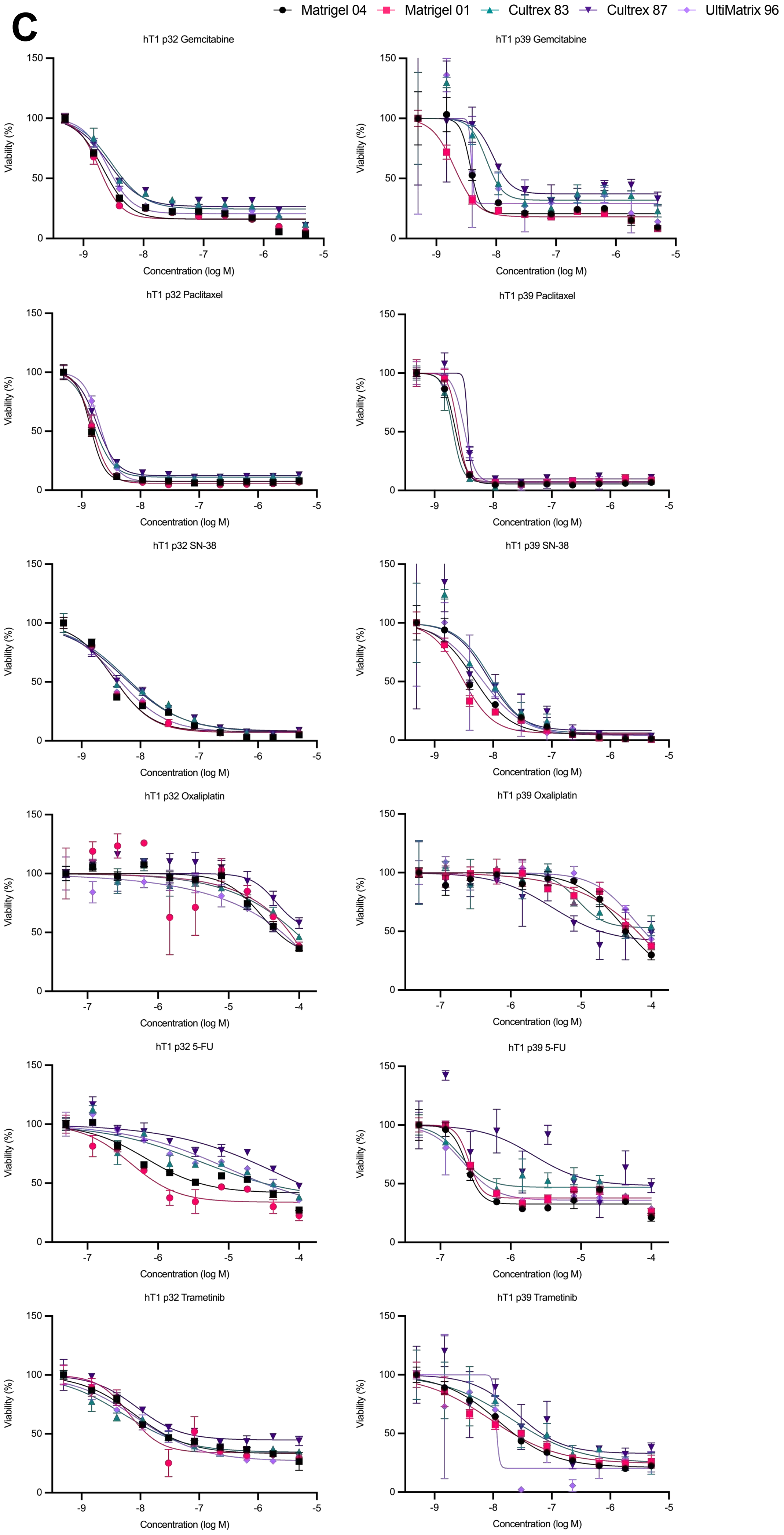

Supplemental Figure 4C

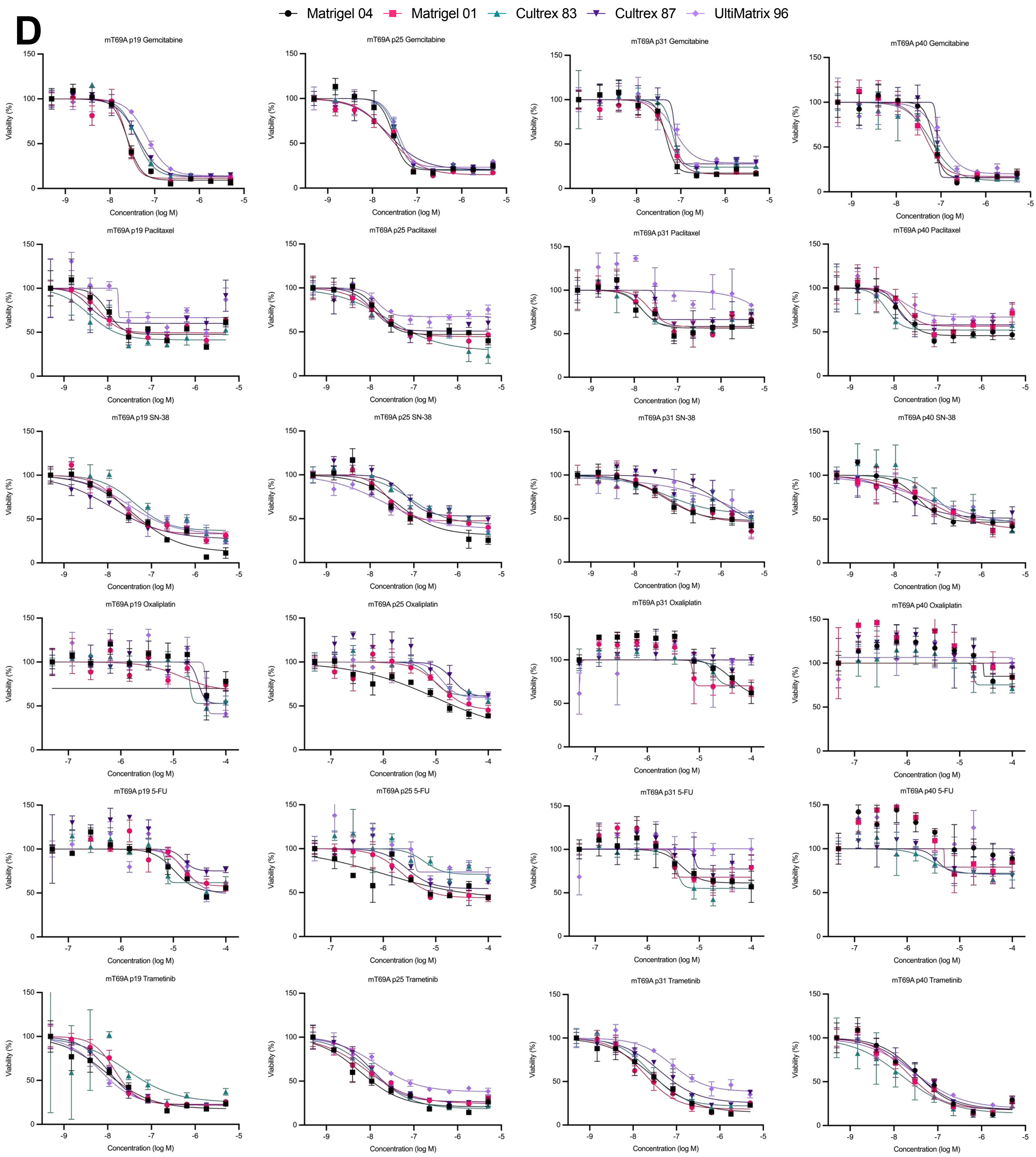

Supplemental Figure 4D

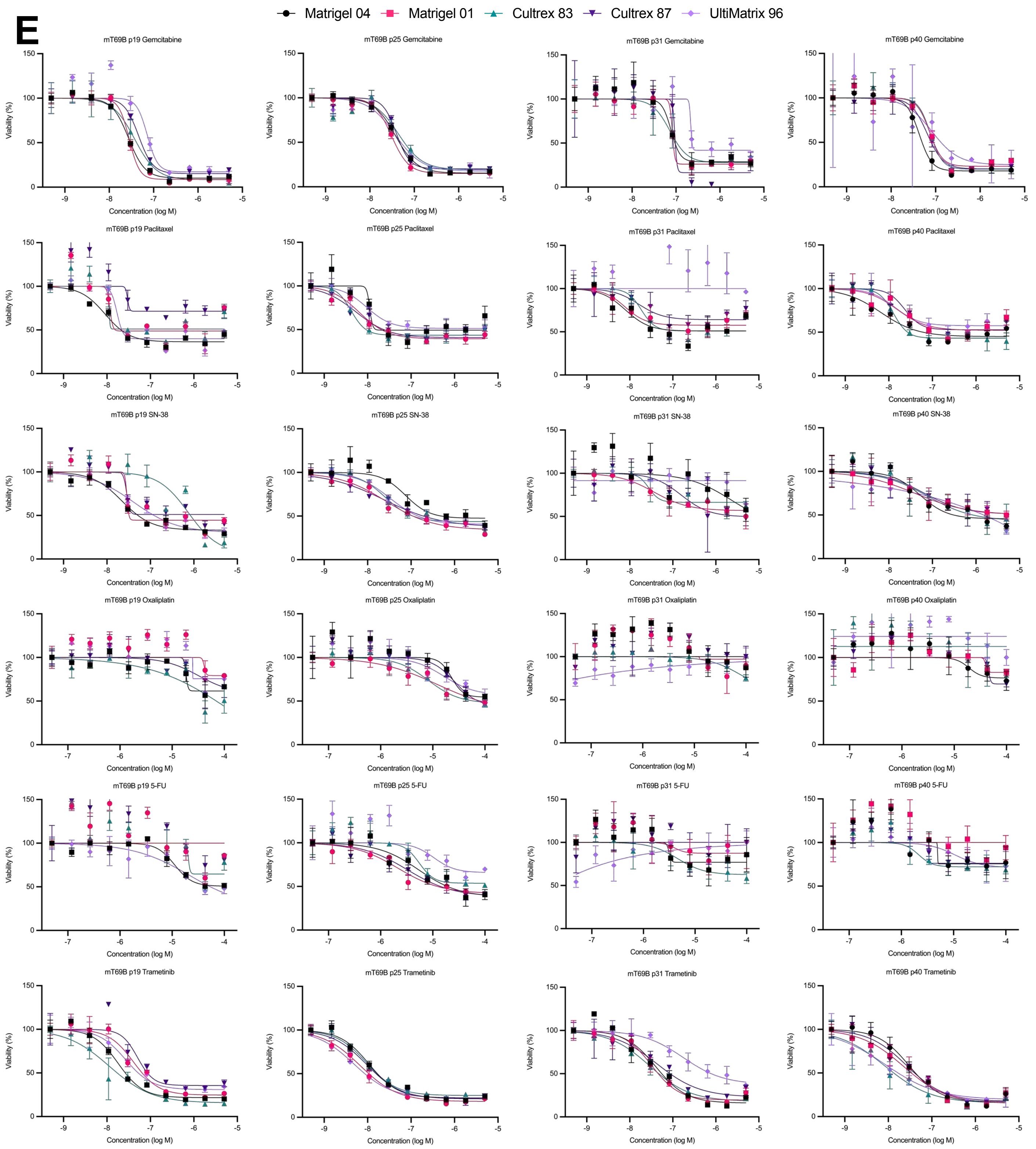

Supplemental Figure 4E

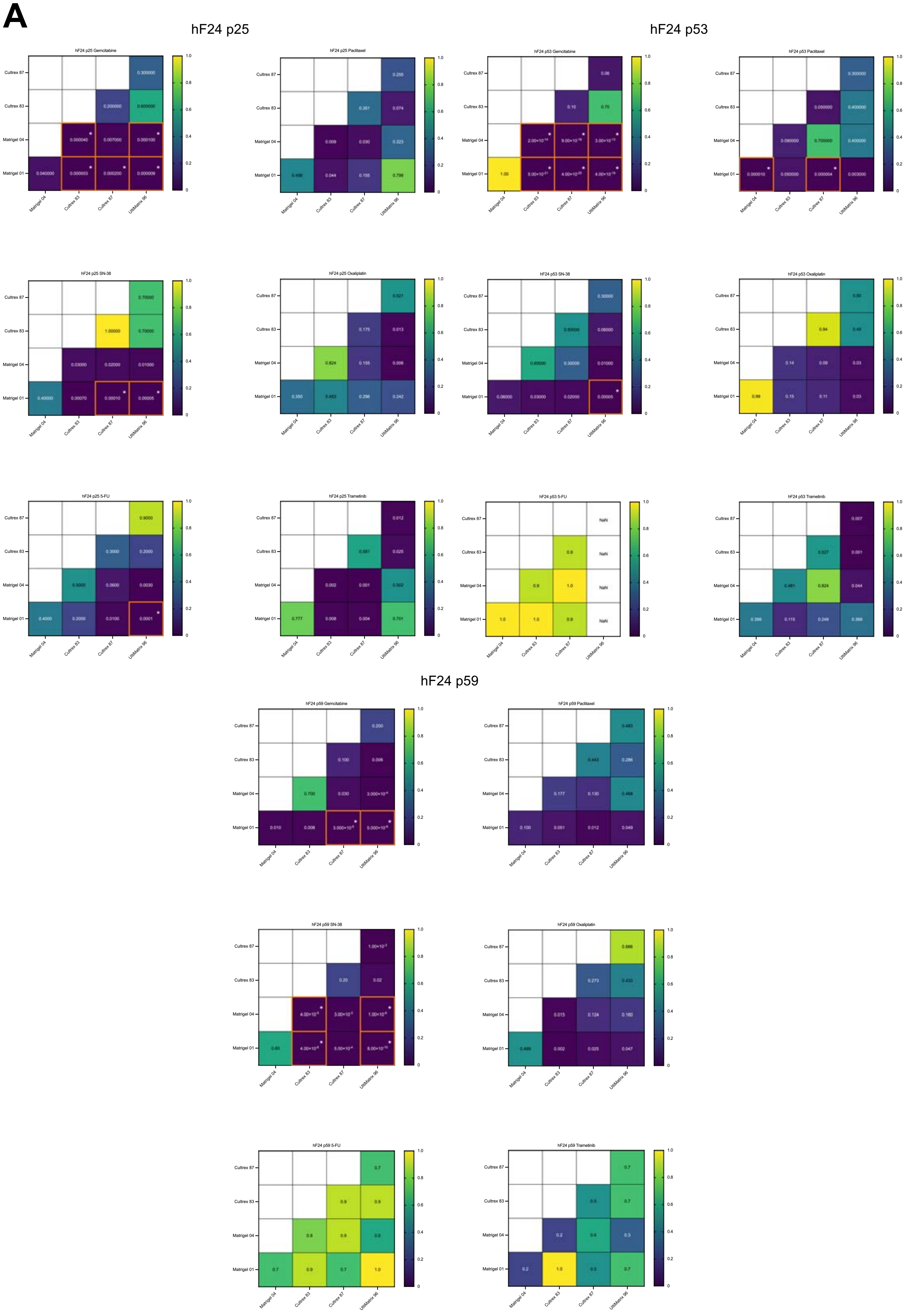

Supplemental Figure 5A – IC50

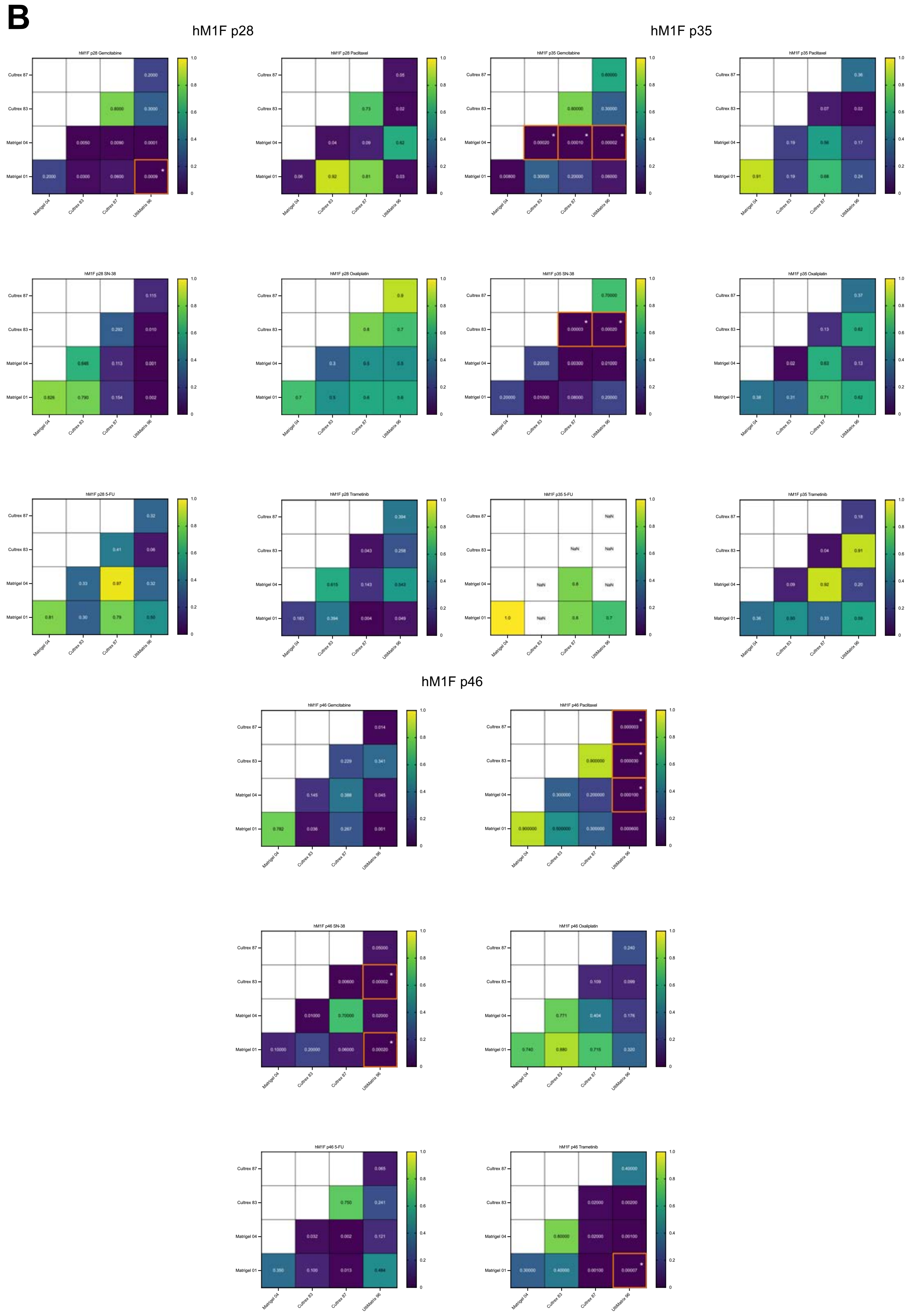

Supplemental Figure 5B – IC50

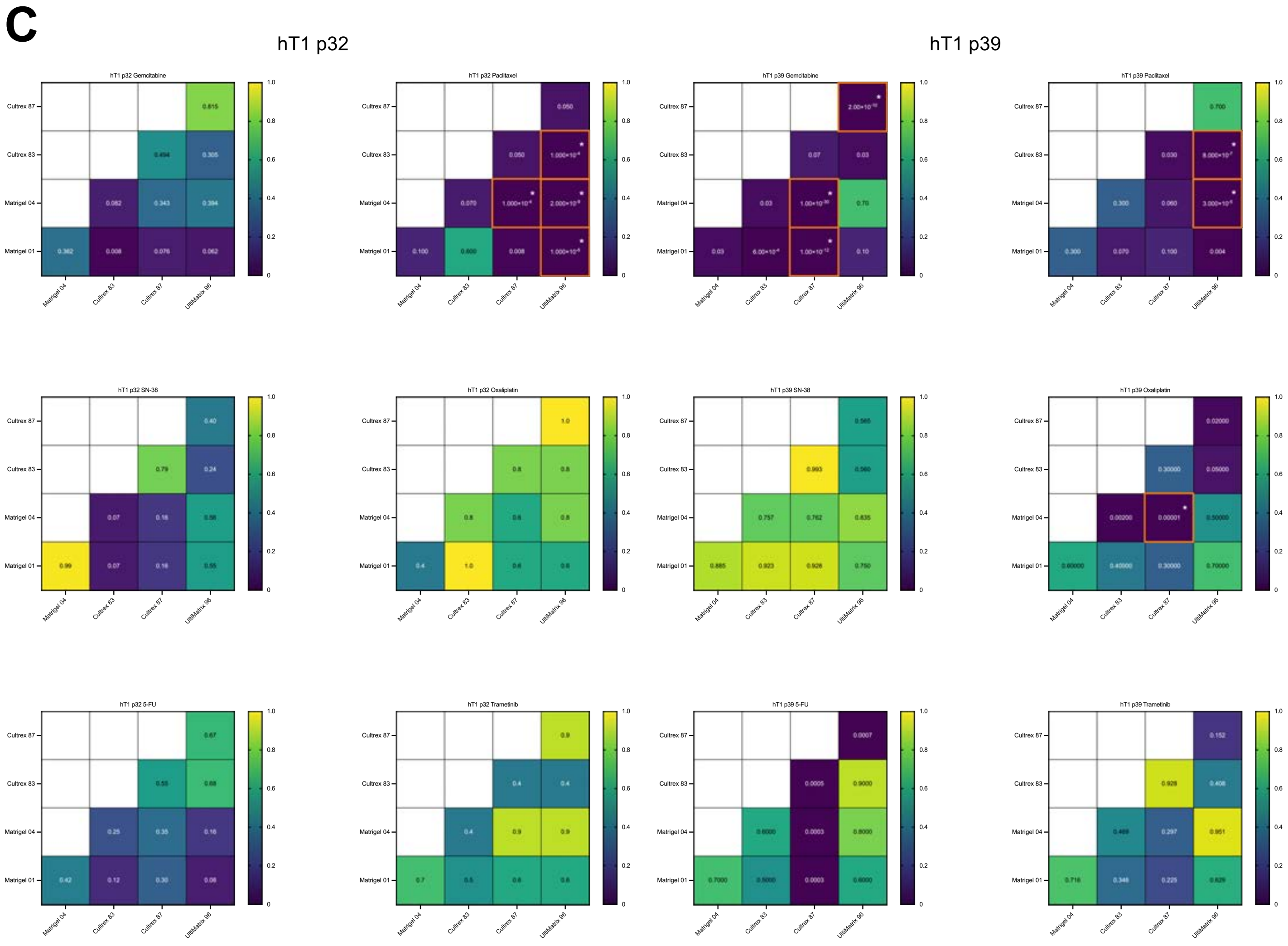

Supplemental Figure 5C – IC50

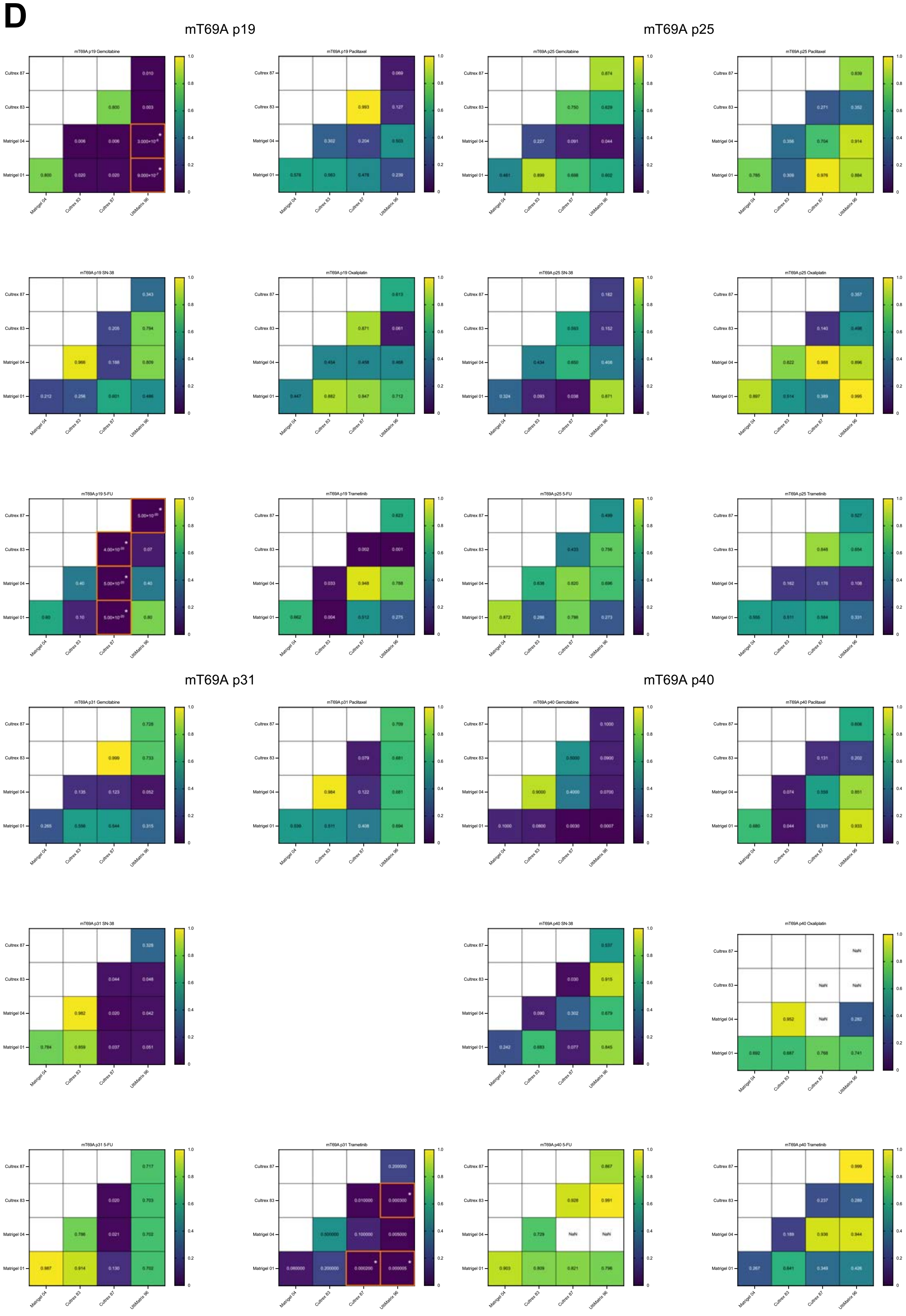

Supplemental Figure 5D – IC50

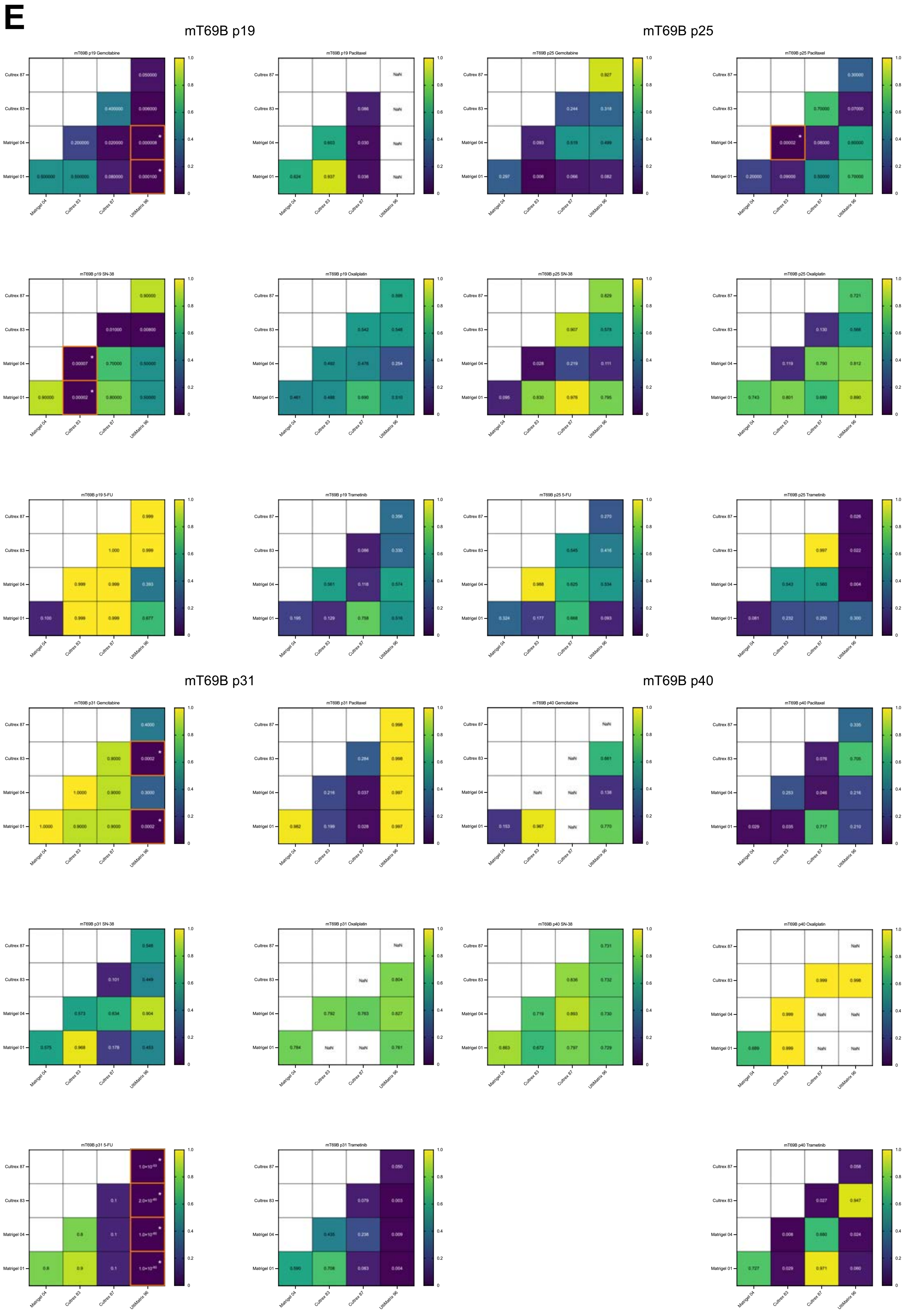

Supplemental Figure 5E – IC50

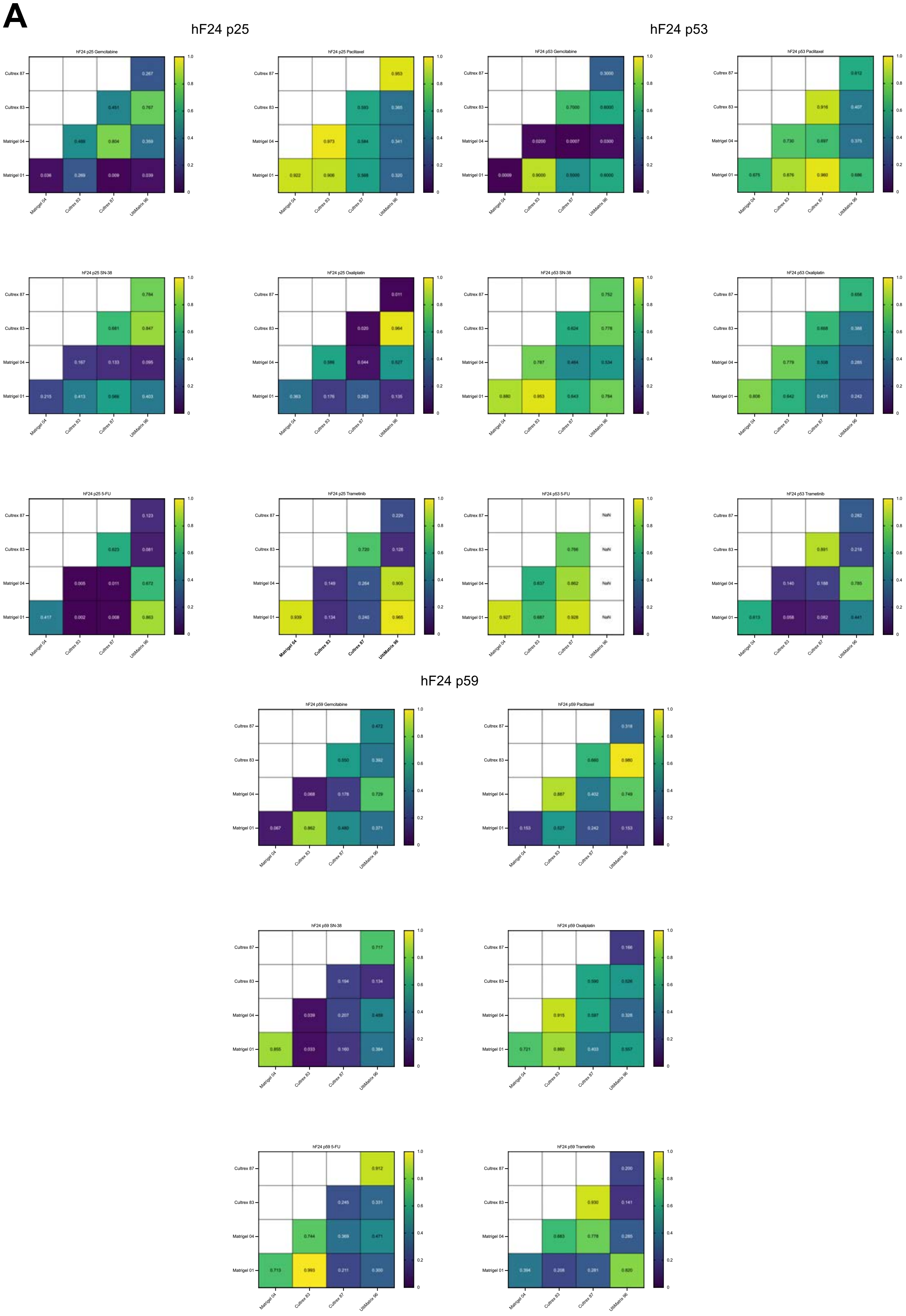

Supplemental Figure 6A – Hill Slope

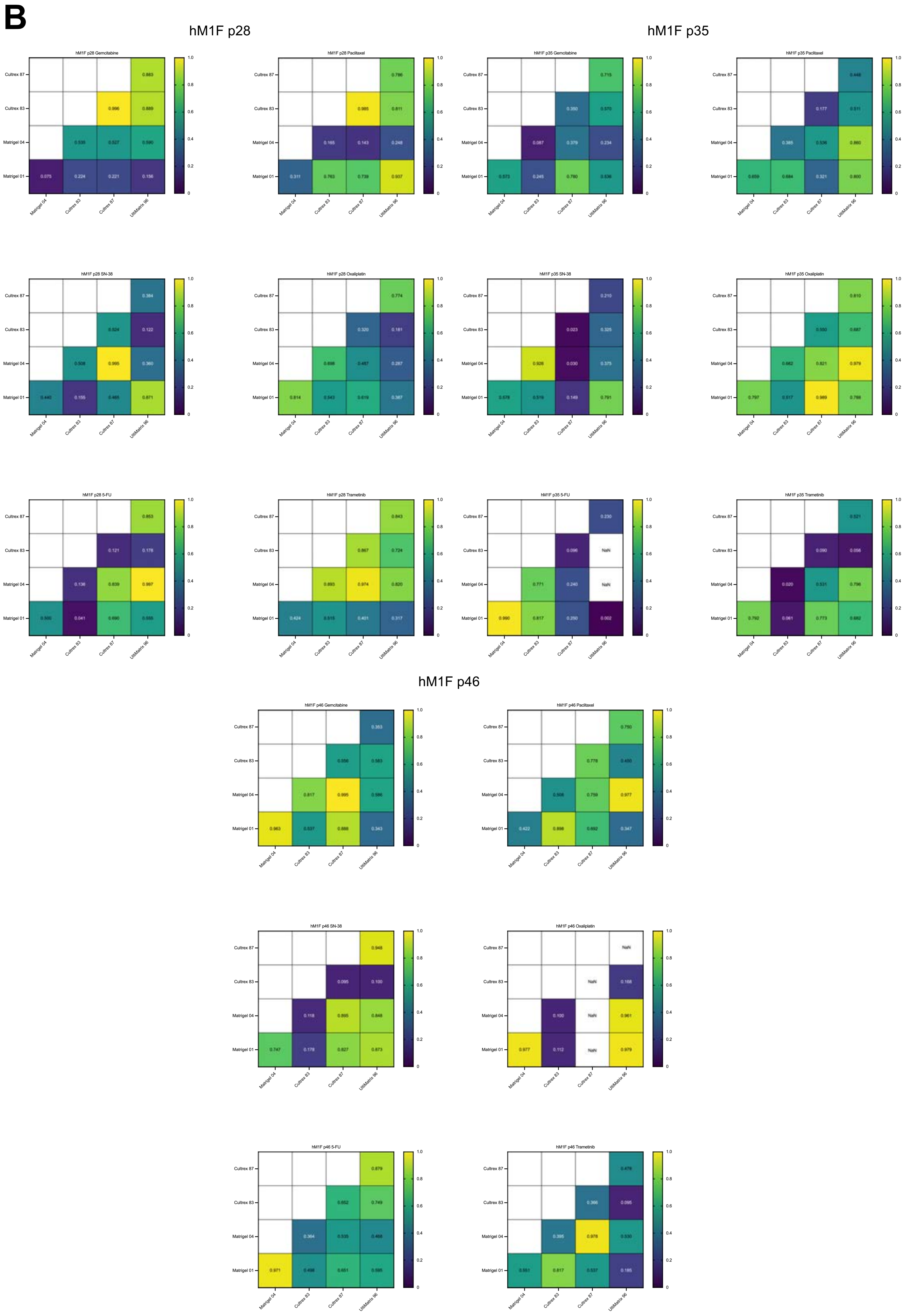

Supplemental Figure 6B – Hill Slope

C

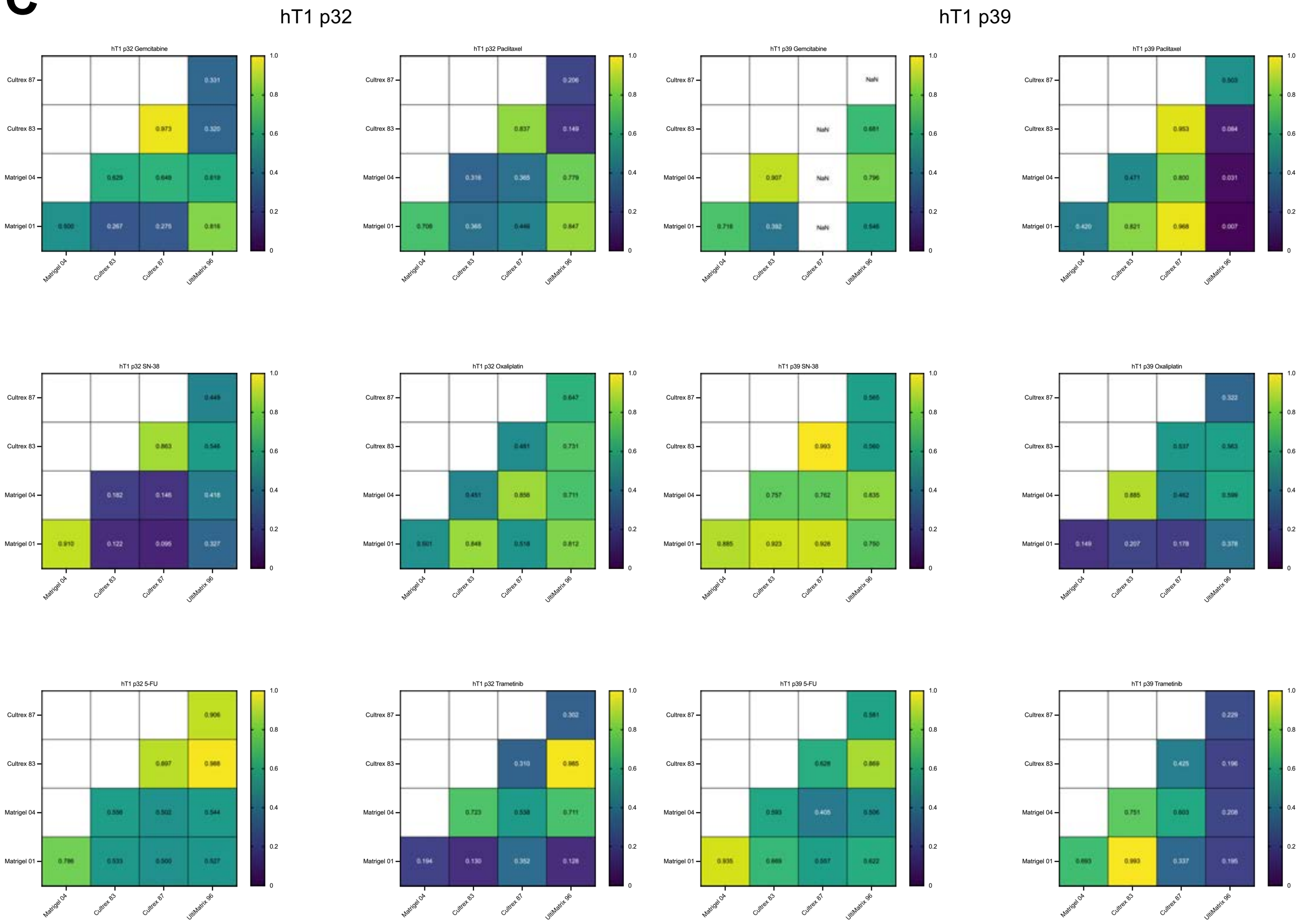

Supplemental Figure 6C – Hill Slope

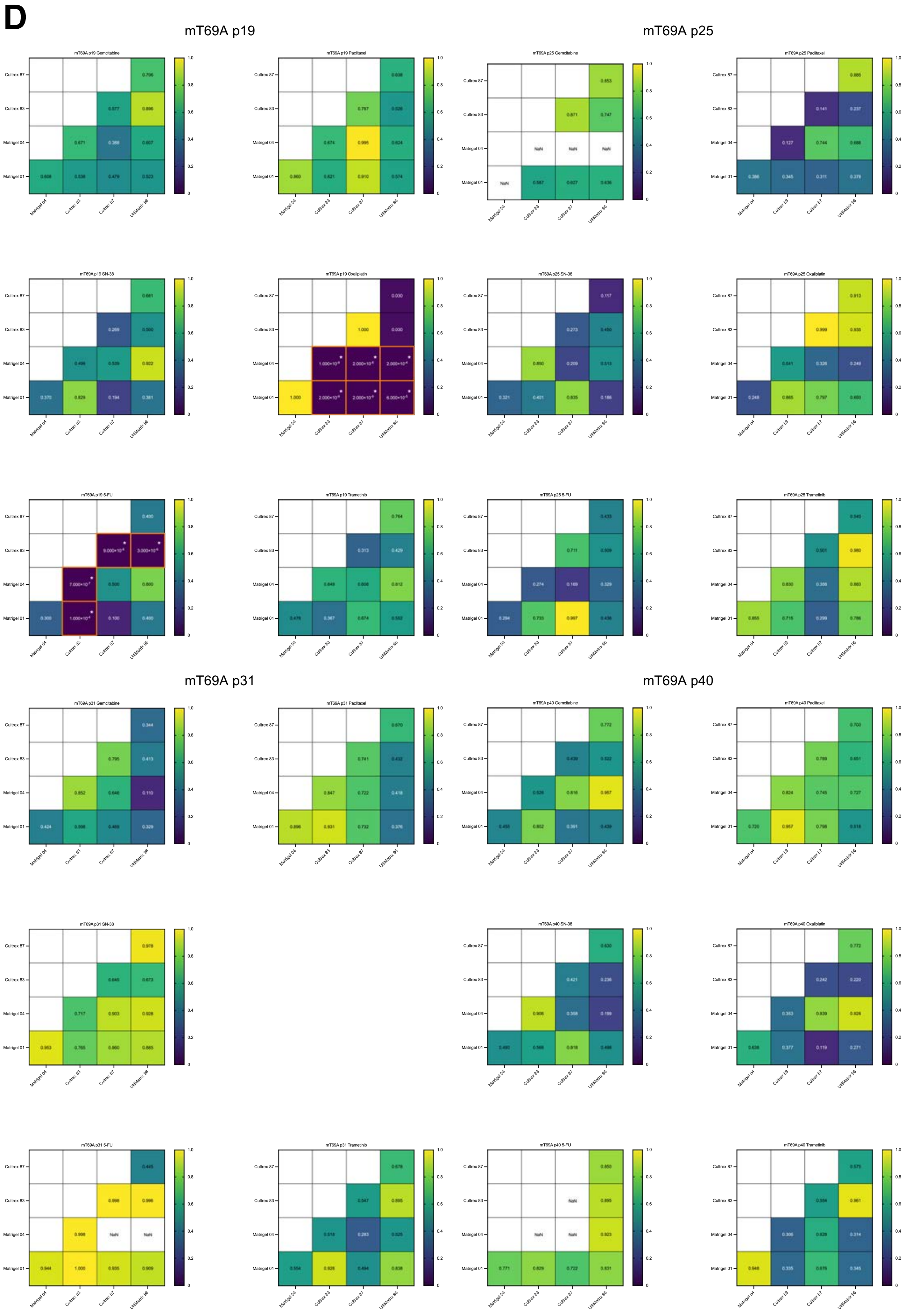

Supplemental Figure 6D – Hill Slope

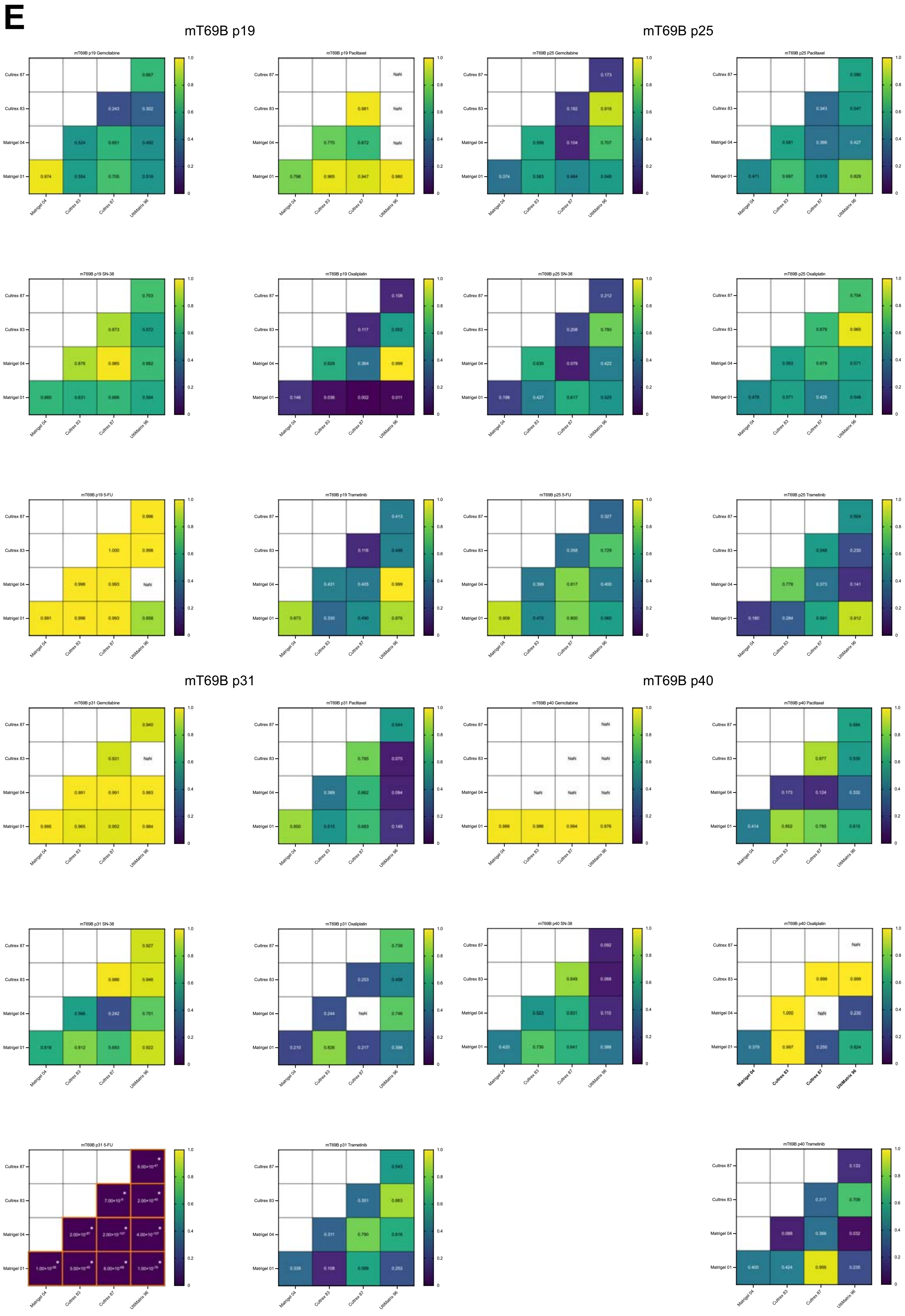

Supplemental Figure 6E – Hill Slope

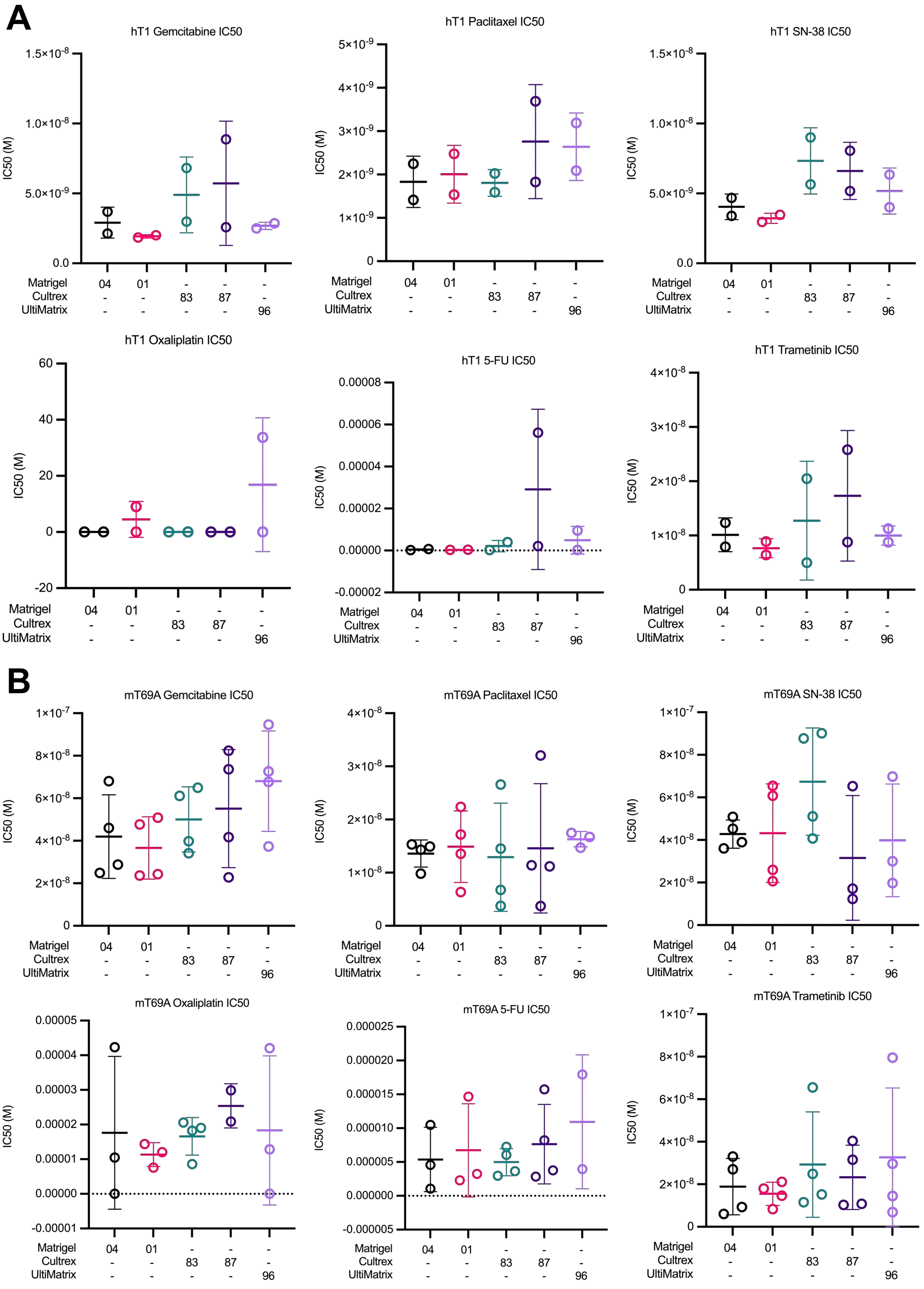

Supplemental Figure 7A-B

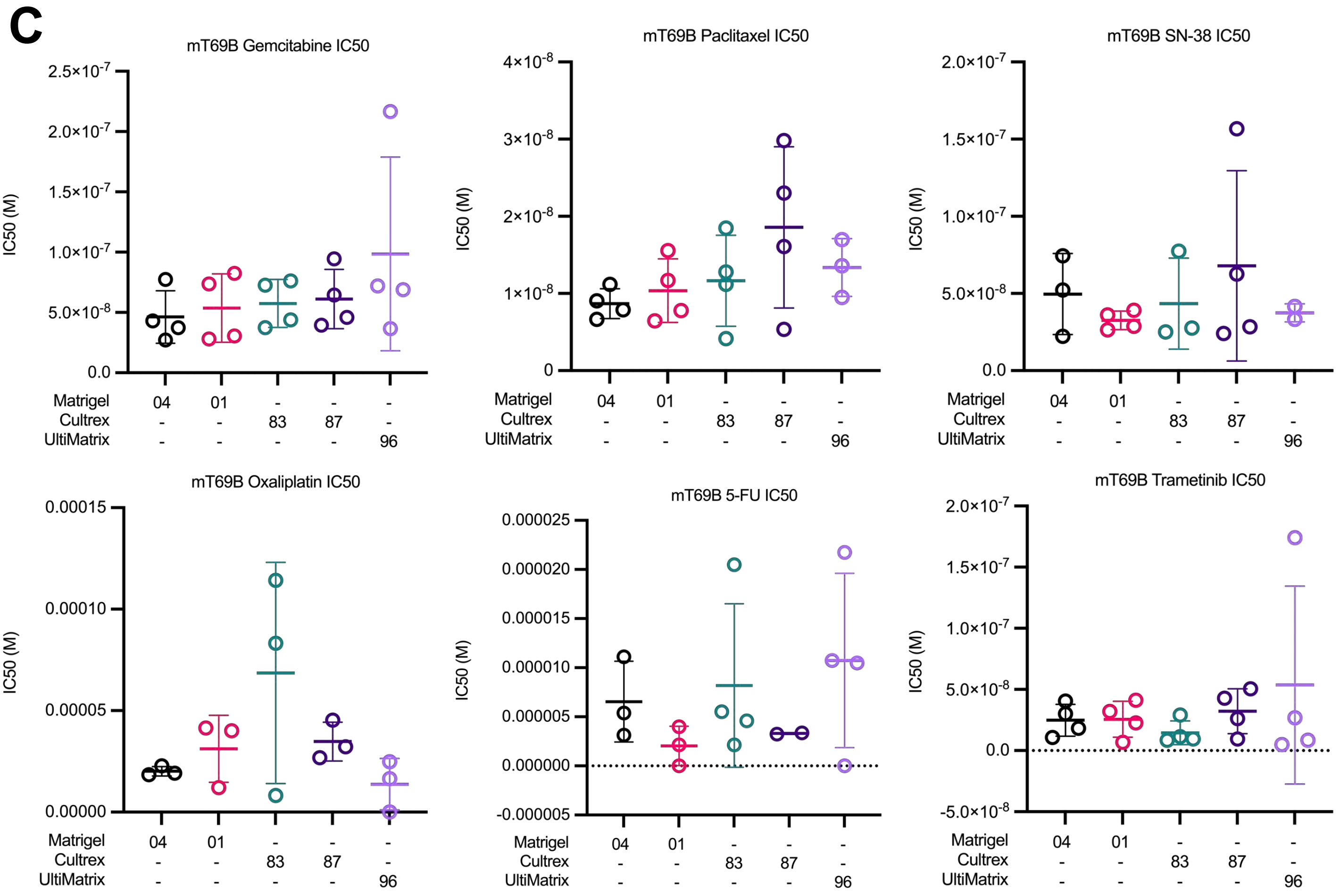

Supplemental Figure 7C

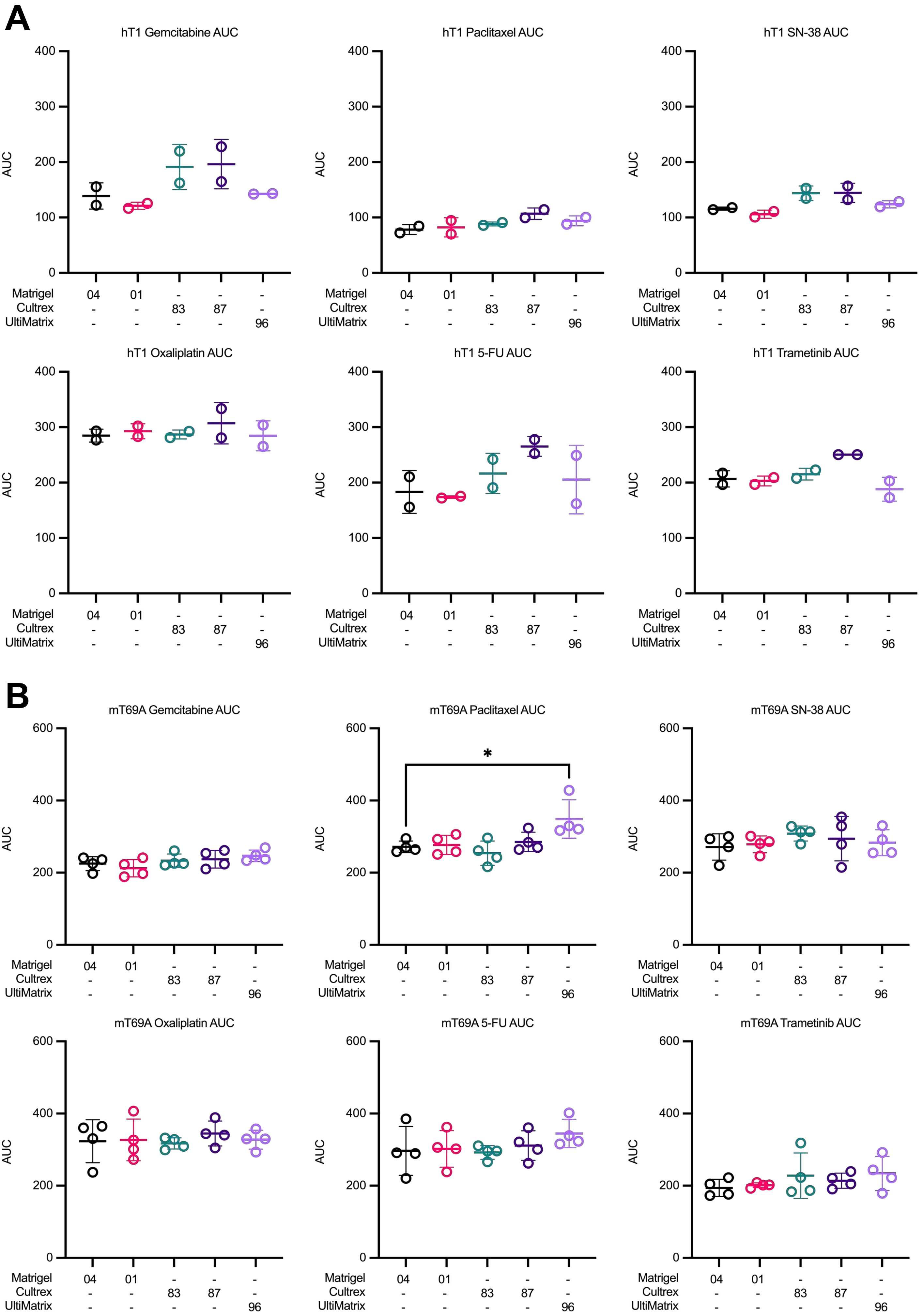

Supplemental Figure 8A-B

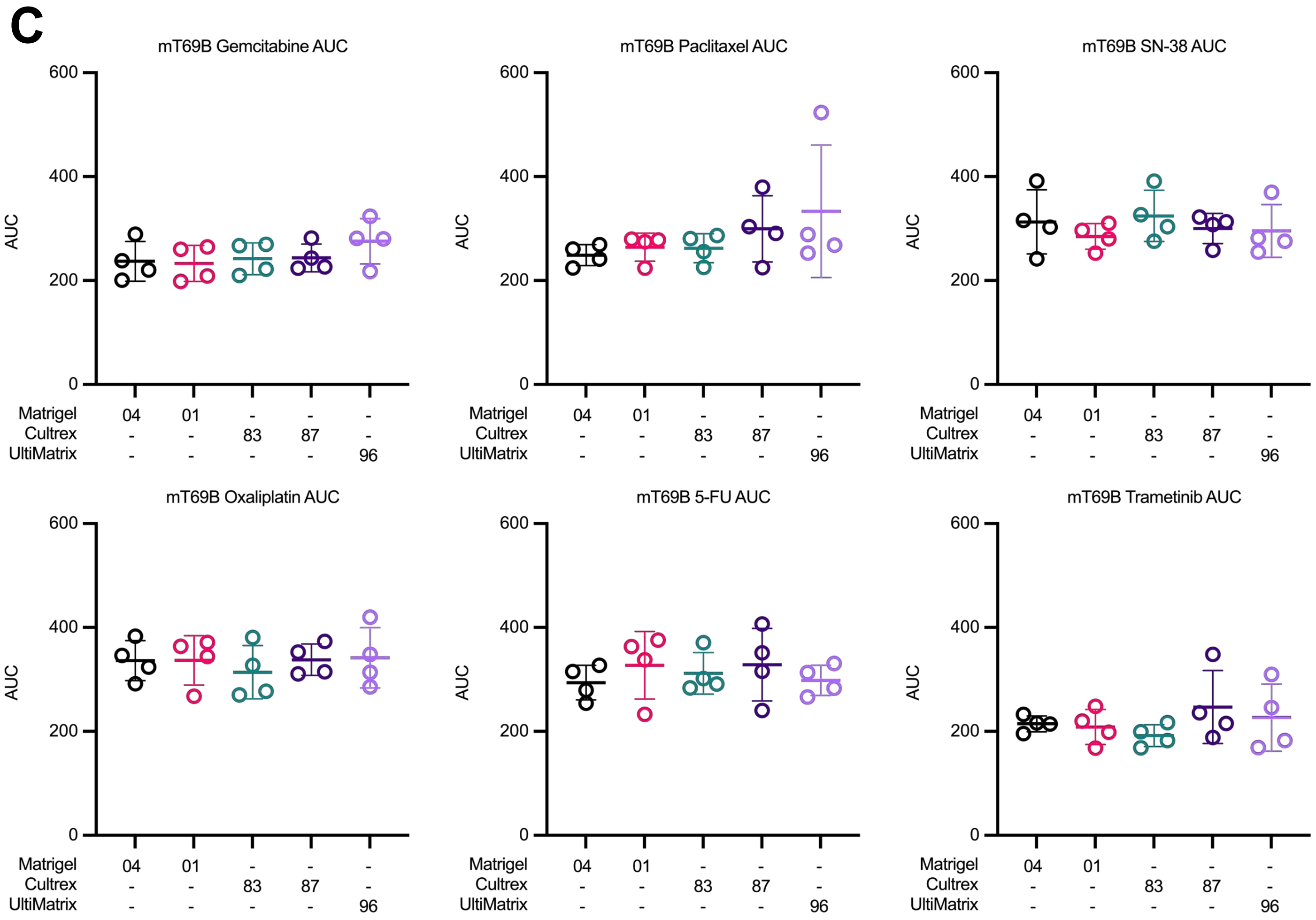

Supplemental Figure 8C

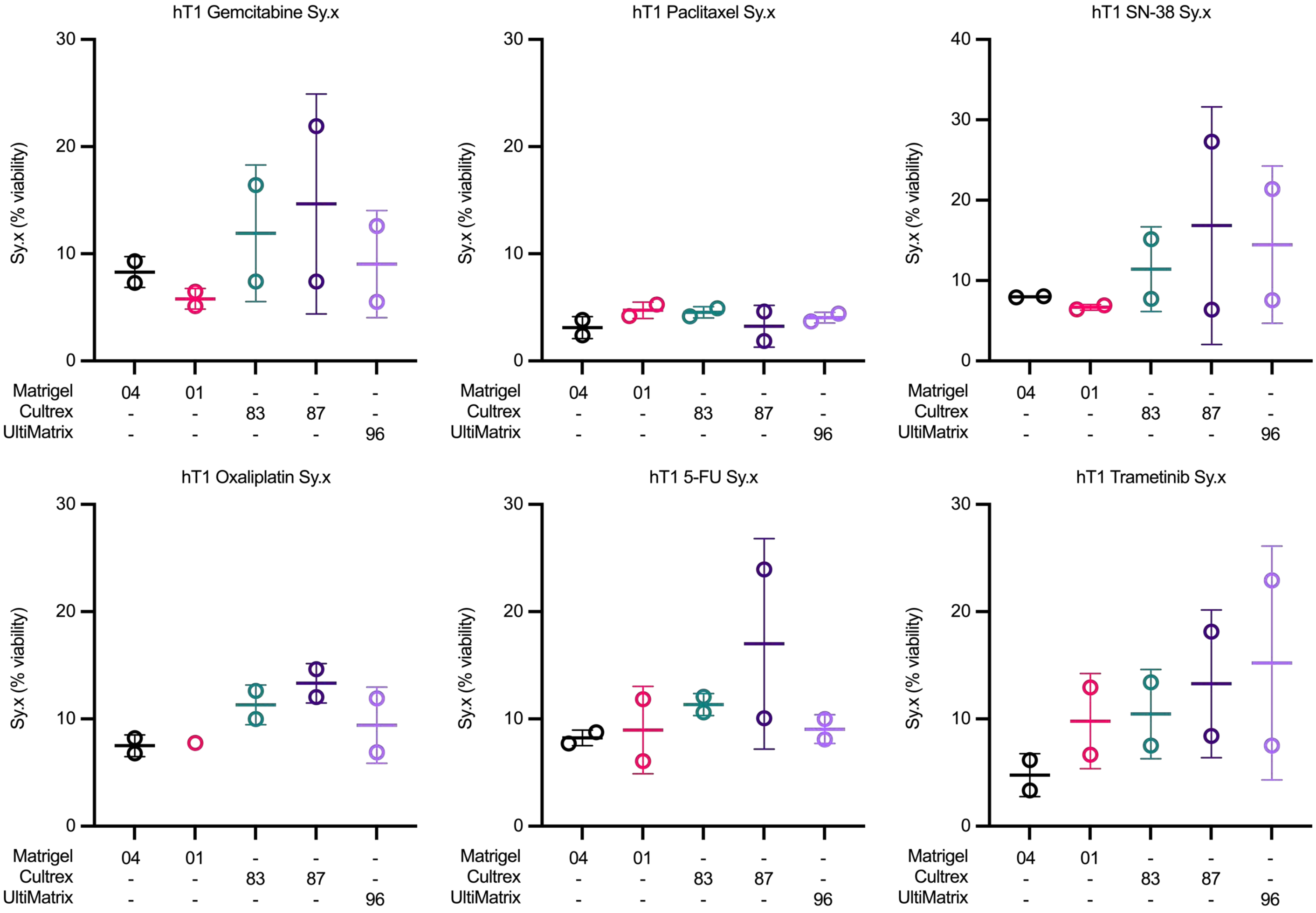

Supplemental Figure 9

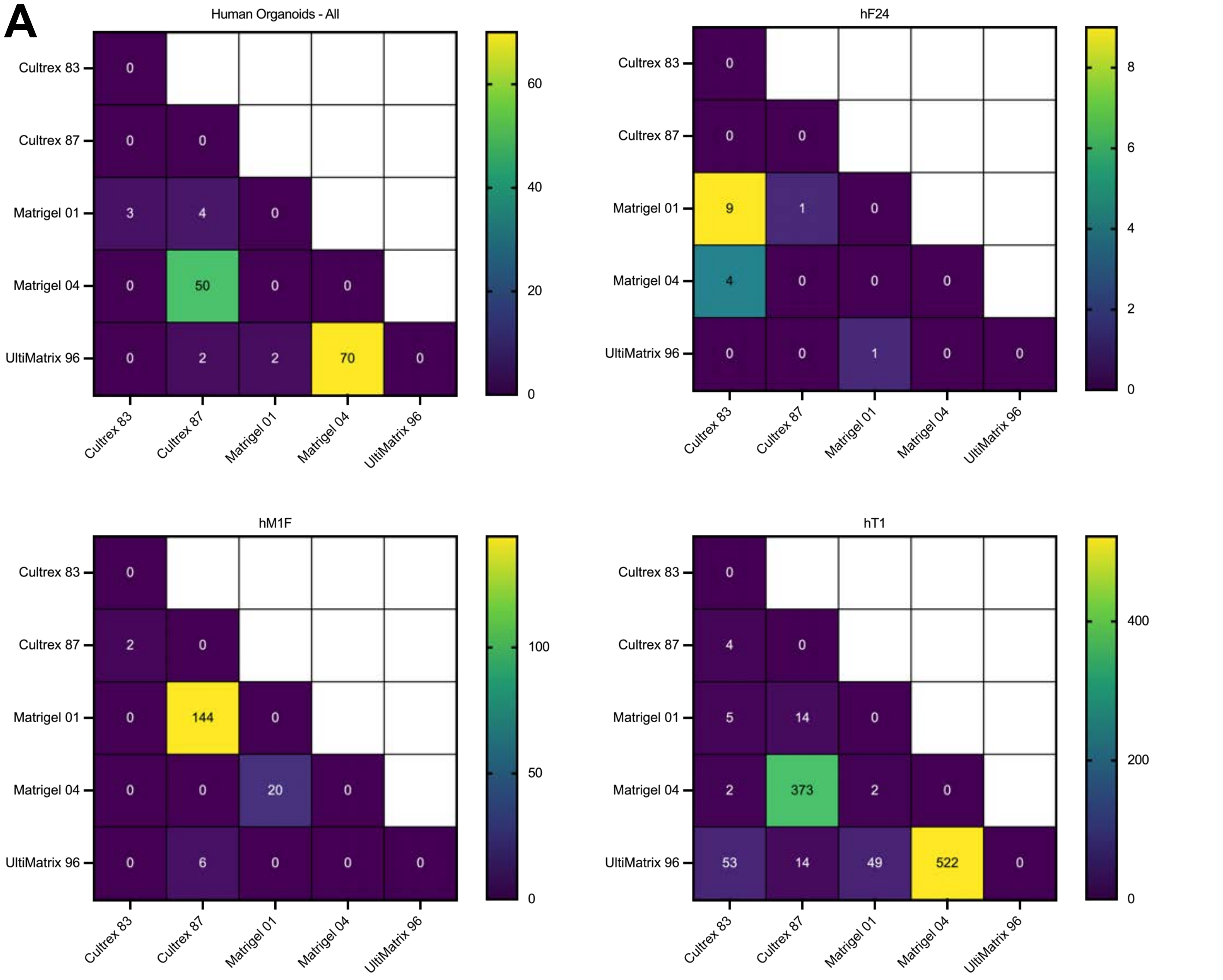

Cells labeled by number of differentially expressed genes (abs(lfc)>1 and FDR ≤ 0.05)

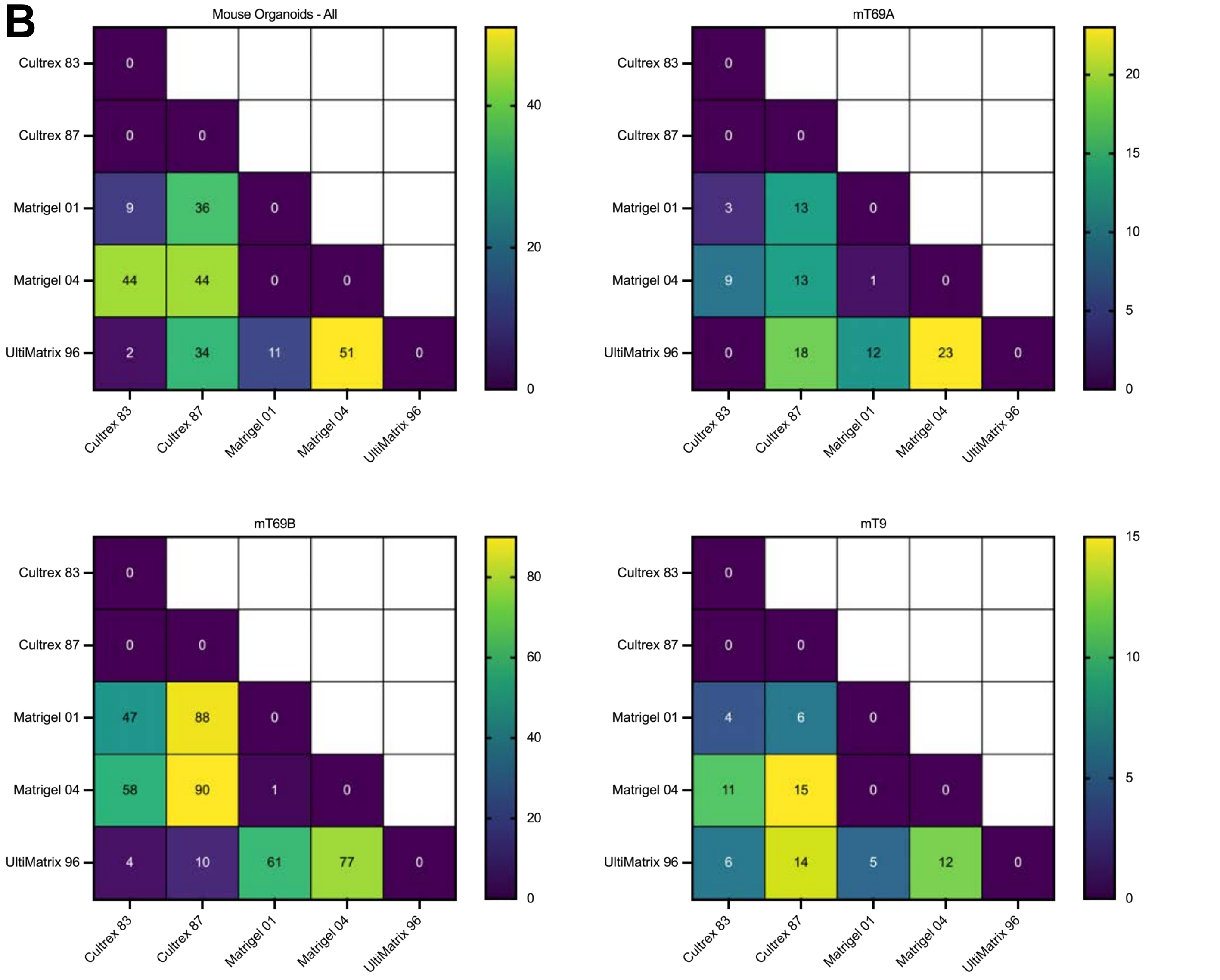

Cells labeled by number of differentially expressed genes (abs(lfc)>1 and FDR ≤ 0.05)

Supplemental Figure 10A-B

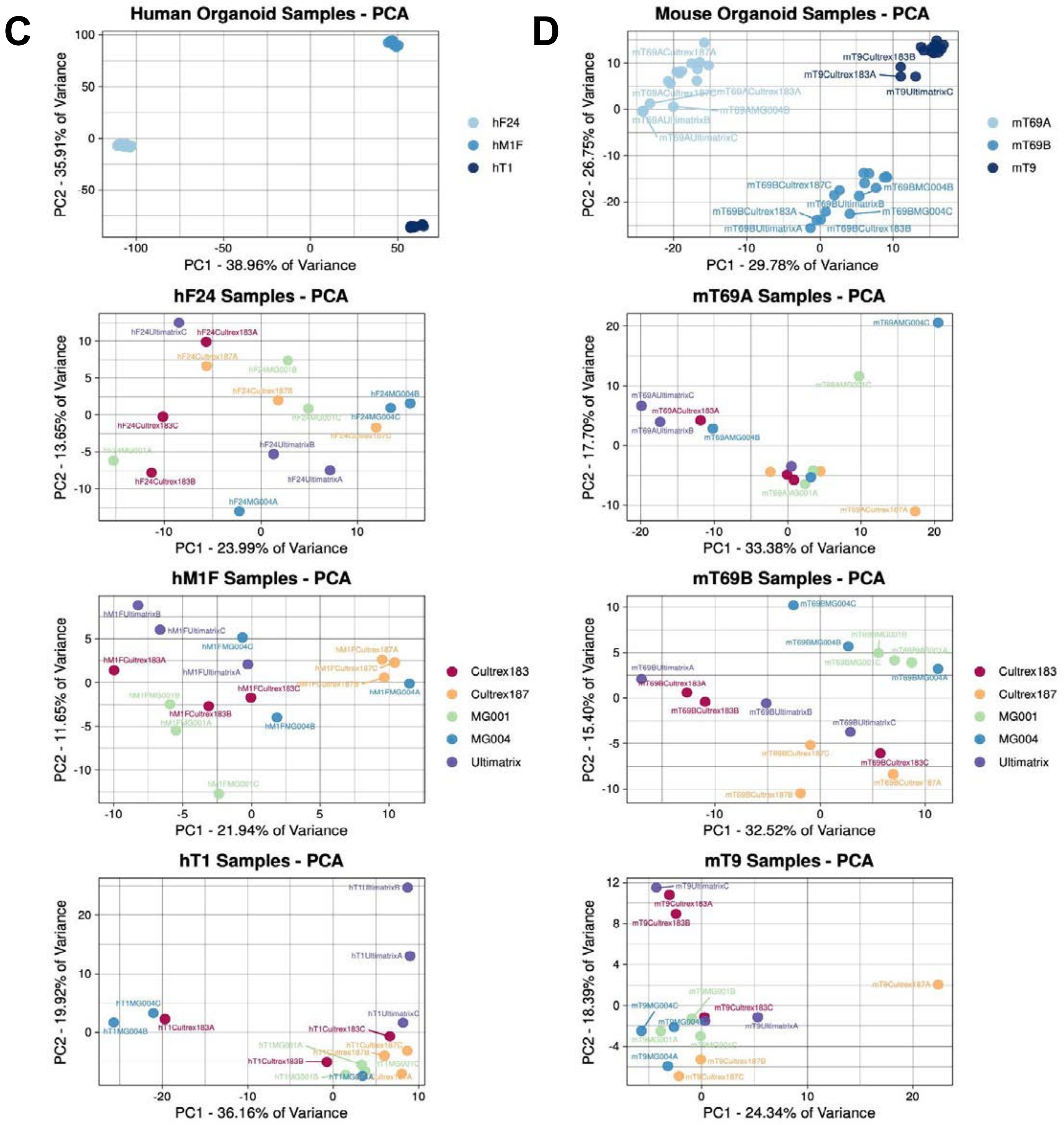

Supplemental Figure 10C-D

E

Expression Heatmap of All Genes
Z-Scores of Human Organoid Normalized Counts

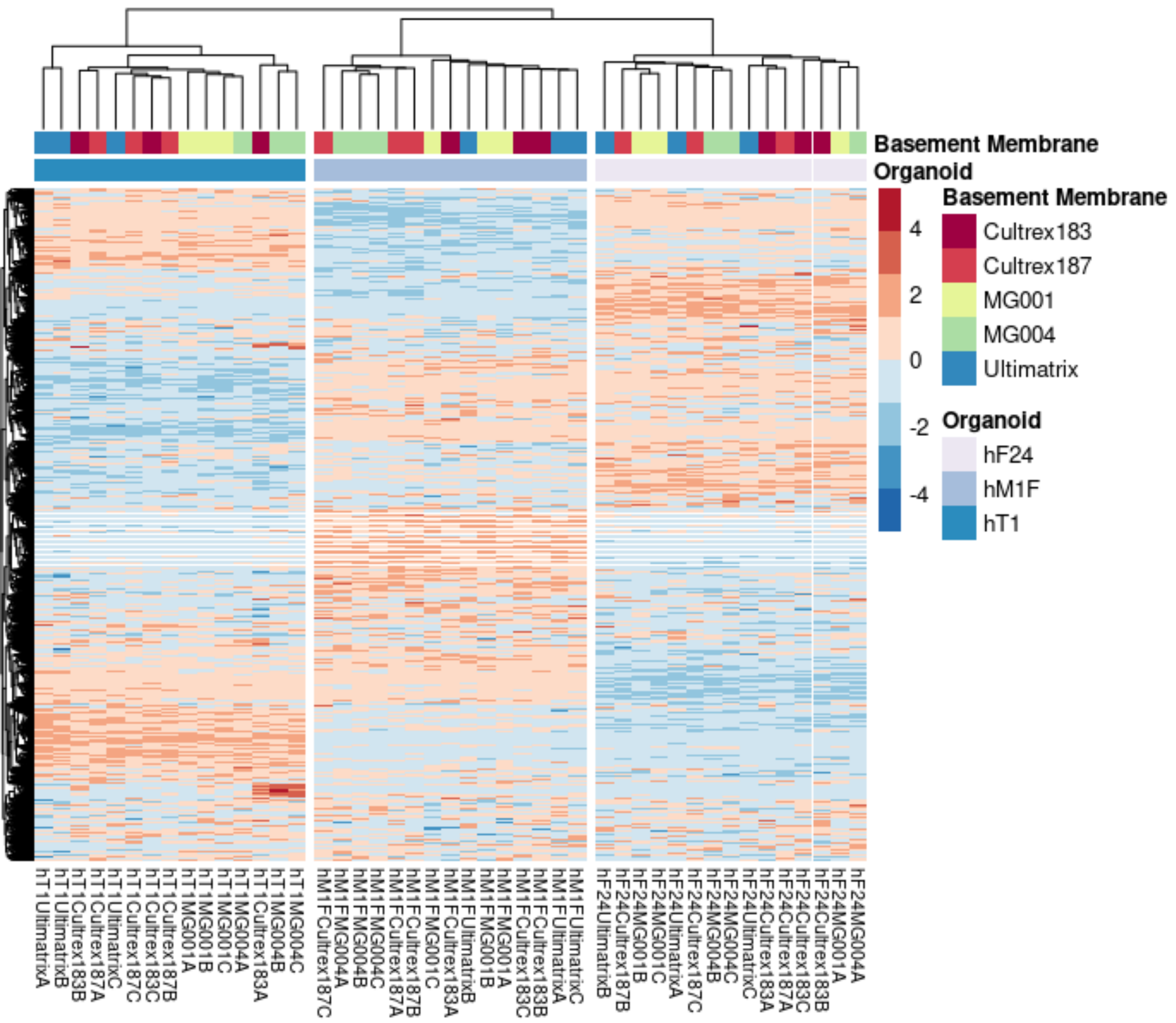

F

Expression Heatmap of All Genes
Z-Scores of Mouse Organoid Normalized Counts

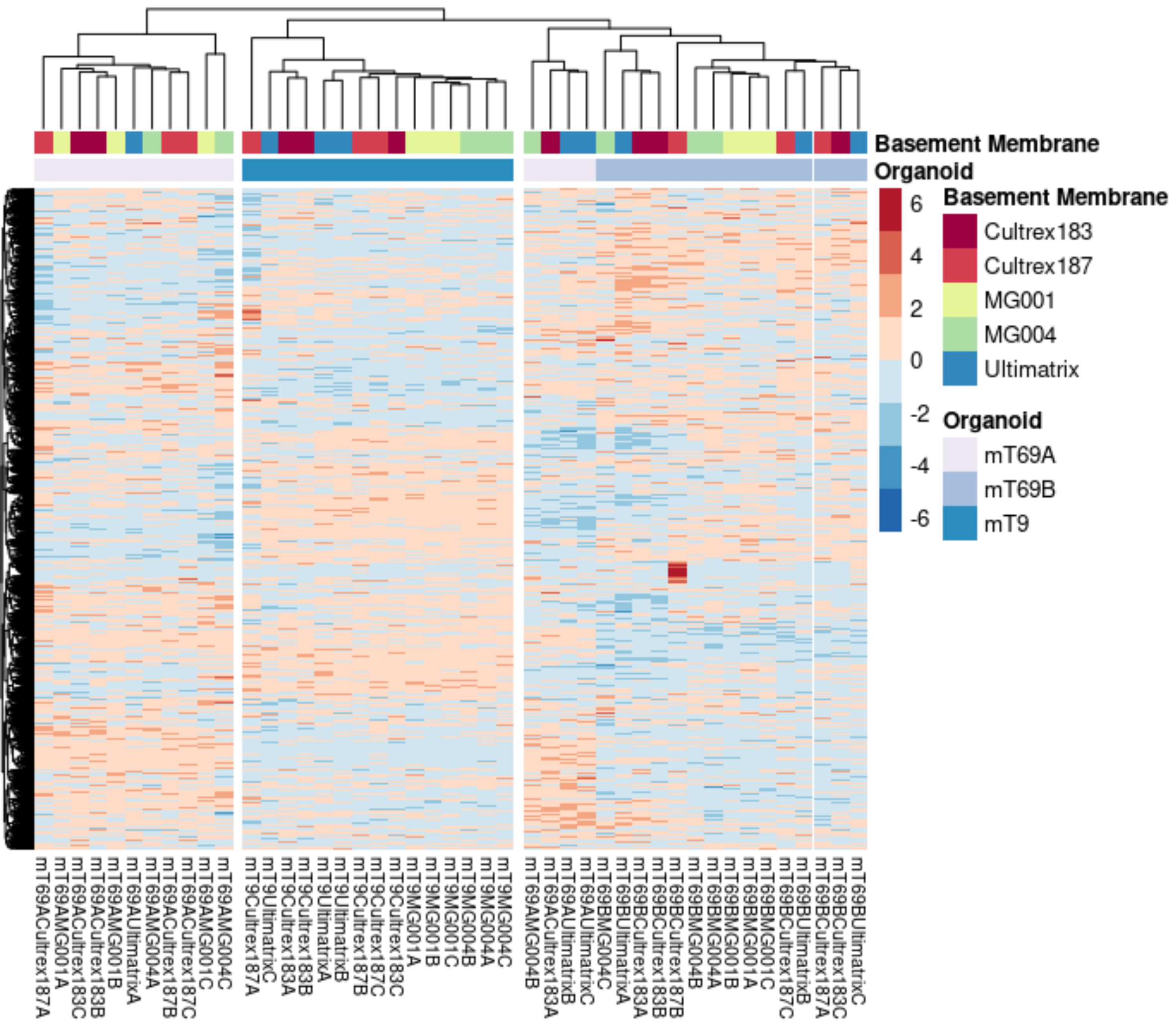

Supplemental Figure 10E-F

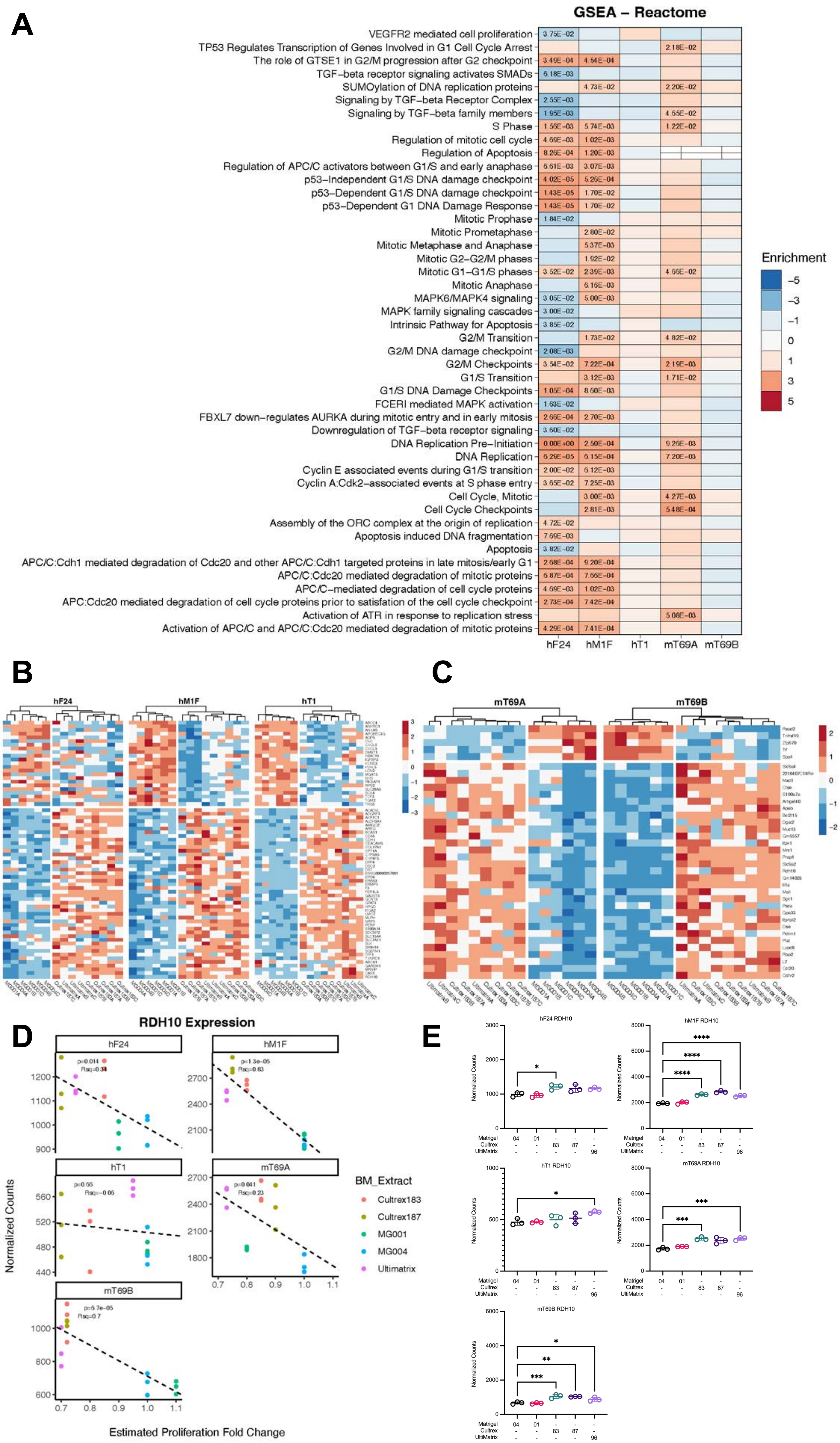

Supplemental Figure 11

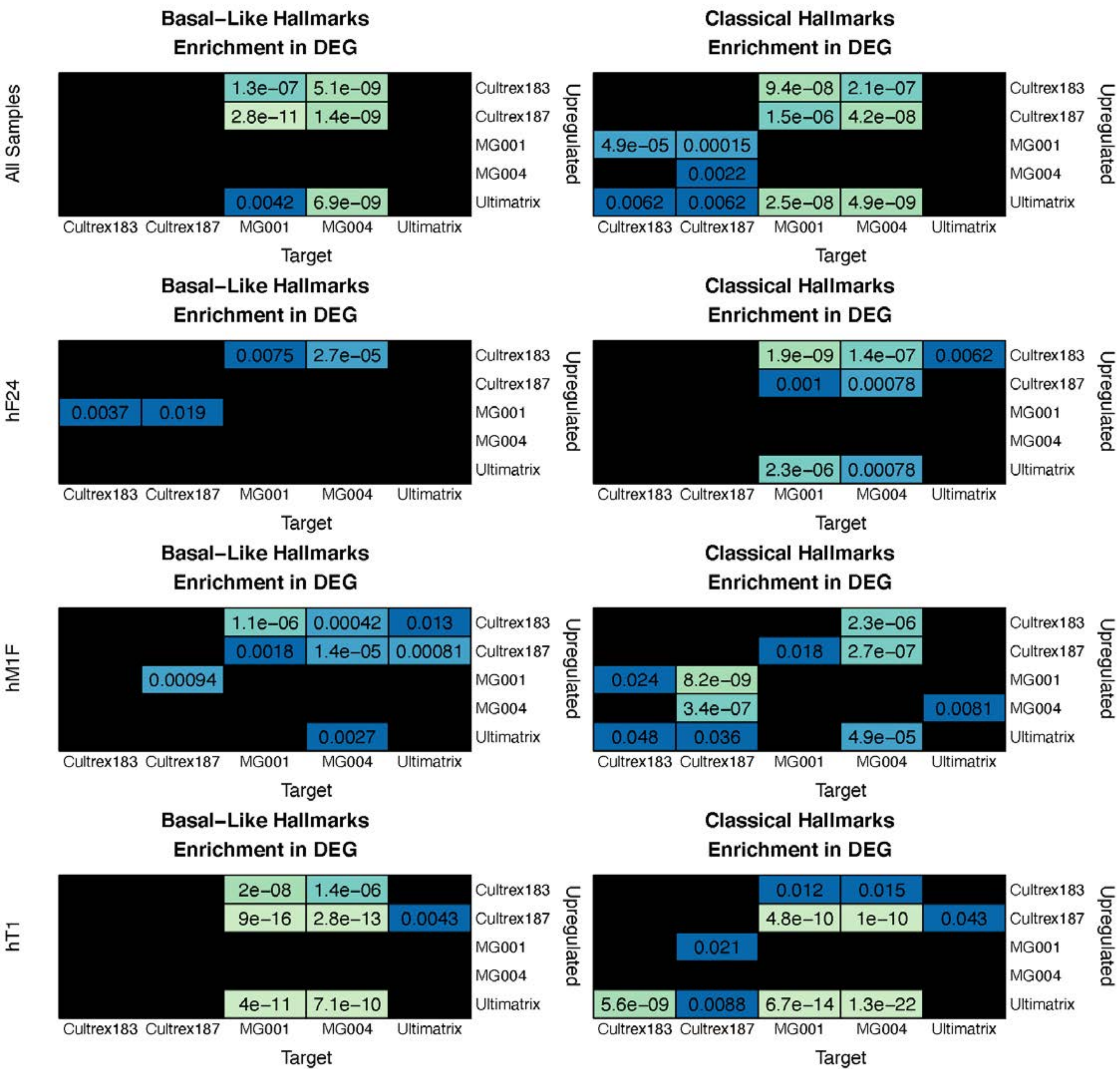

Supplemental Figure 12

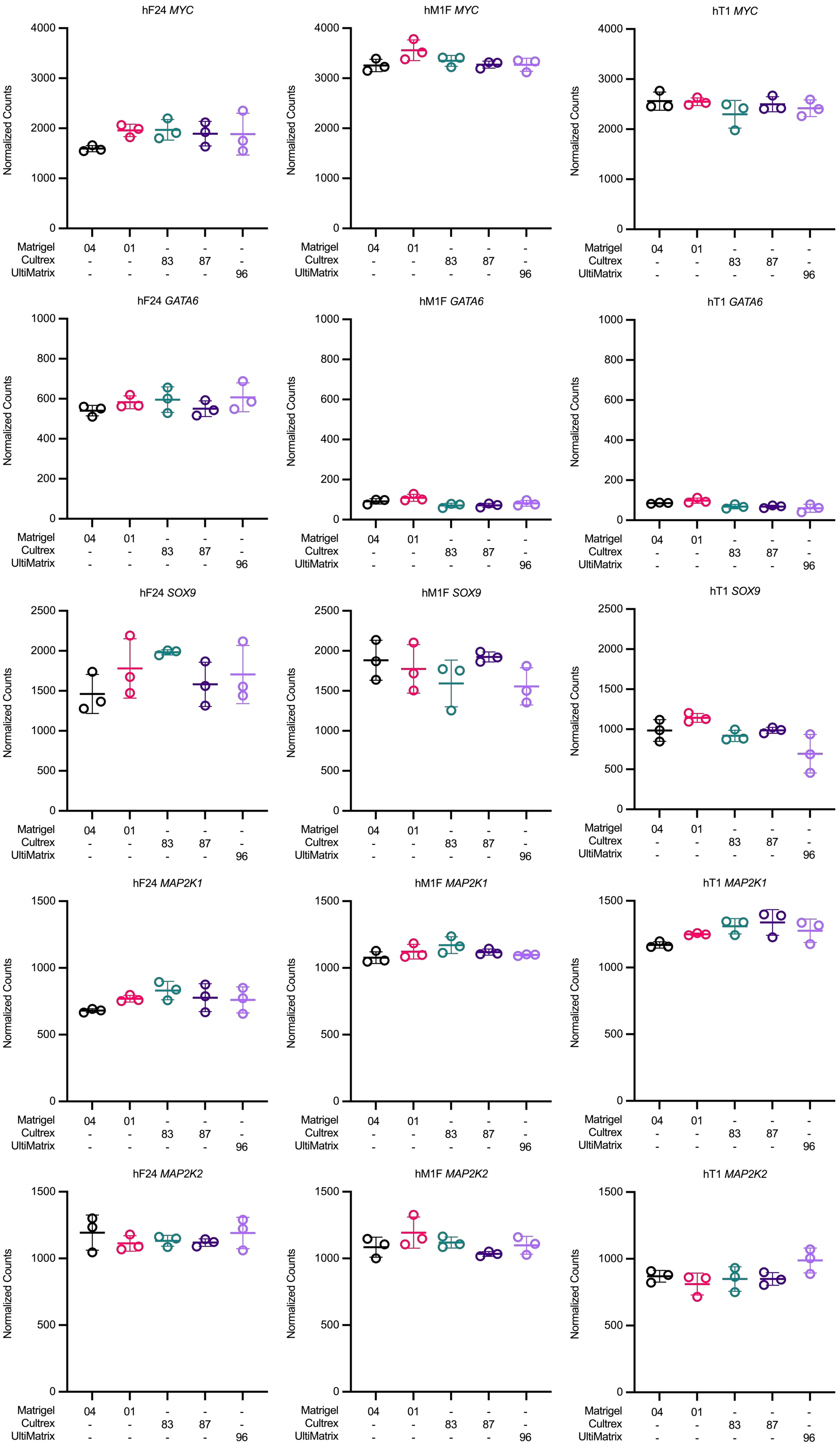

Supplemental Figure 13

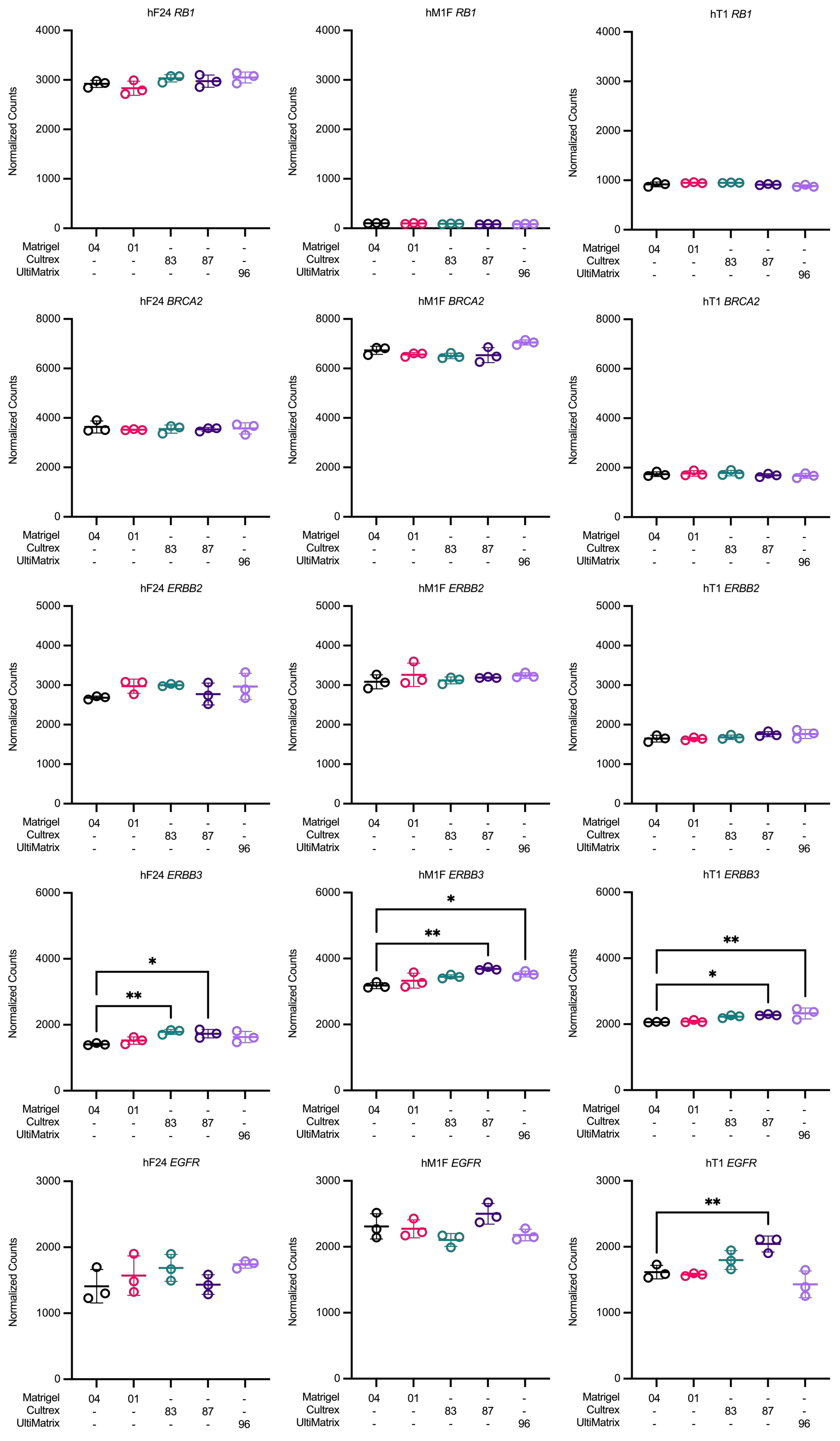

Supplemental Figure 13

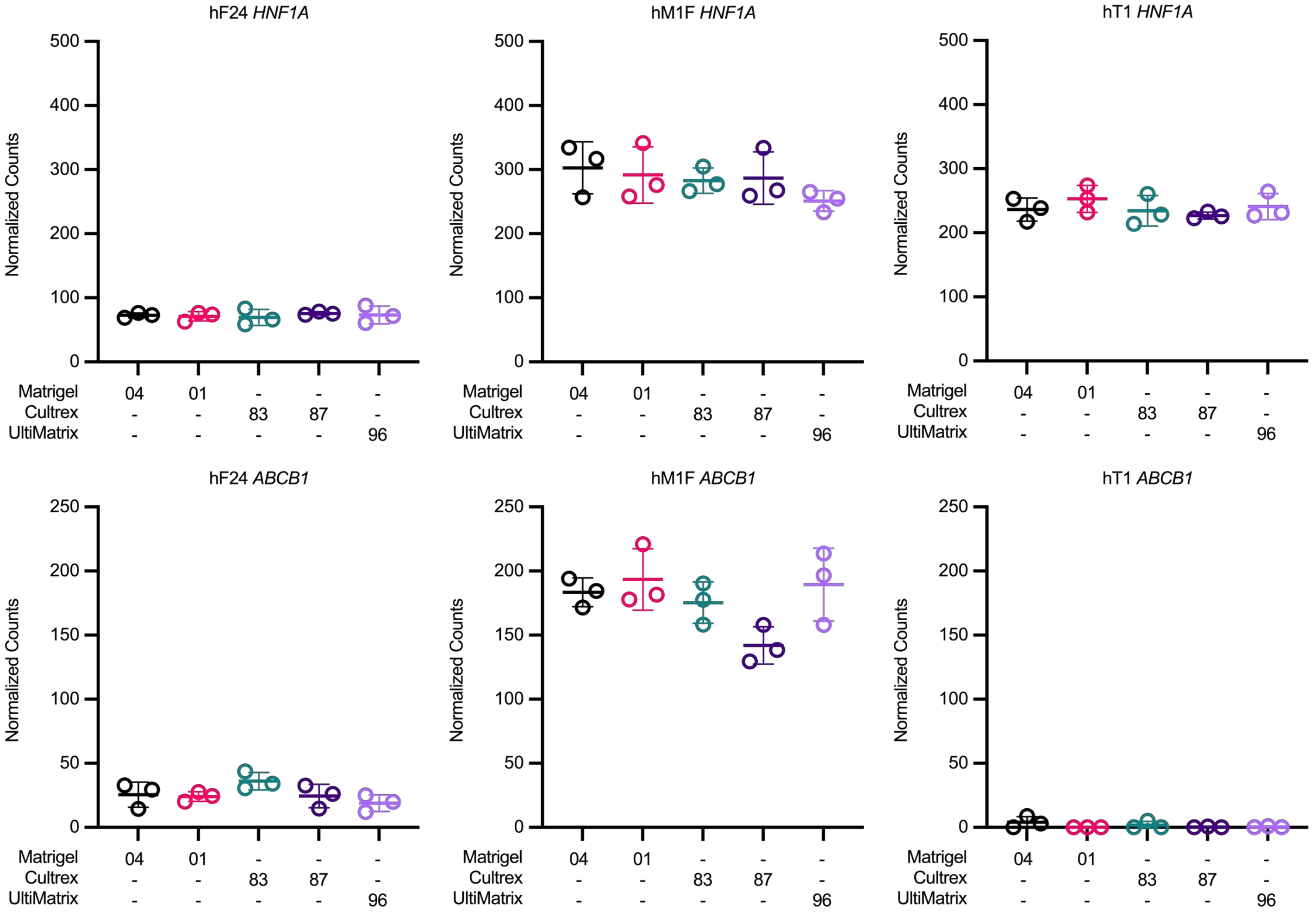

Supplemental Figure 13
